# Focus on Over-the-Counter Drugs' Misuse: A Systematic Review on Antihistamines, Cough Medicines, and Decongestants

**DOI:** 10.3389/fpsyt.2021.657397

**Published:** 2021-05-07

**Authors:** Fabrizio Schifano, Stefania Chiappini, Andrea Miuli, Alessio Mosca, Maria Chiara Santovito, John M. Corkery, Amira Guirguis, Mauro Pettorruso, Massimo Di Giannantonio, Giovanni Martinotti

**Affiliations:** ^1^Psychopharmacology, Drug Misuse and Novel Psychoactive Substances Research Unit, School of Life and Medical Sciences, University of Hertfordshire, Hatfield, United Kingdom; ^2^Department of Neuroscience, Imaging and Clinical Sciences, “G. D'Annunzio” University, Chieti, Italy; ^3^Swansea University Medical School, Institute of Life Sciences 2, Swansea University, Swansea, United Kingdom

**Keywords:** drug abuse, drug misuse, prescription drug misuse, pharming, drug diversion, over the counter drug misuse, addiction, OTC

## Abstract

**Background:** Over the past 20 years or so, the drug misuse scenario has seen the emergence of both prescription-only and over-the-counter (OTC) medications being reported as ingested for recreational purposes. OTC drugs such as antihistamines, cough/cold medications, and decongestants are reportedly the most popular in being diverted and misused.

**Objective:** While the current related knowledge is limited, the aim here was to examine the published clinical data on OTC misuse, focusing on antihistamines (e.g., diphenhydramine, promethazine, chlorpheniramine, and dimenhydrinate), dextromethorphan (DXM)- and codeine-based cough medicines, and the nasal decongestant pseudoephedrine.

**Methods:** A systematic literature review was carried out with the help of Scopus, Web of Science databases, and the related gray literature. For data gathering purposes, both the Preferred Reporting Items for Systematic Reviews and Meta-analyses (PRISMA) and PROSPERO guidelines were followed (PROSPERO identification code CRD42020209261).

**Results:** After completion of the selection, eligibility, and screening phases, some 92 articles were here taken into consideration; case reports, surveys, and retrospective case series analyses were included. Findings were organized according to the specific OTC recorded. Most articles focused here on DXM (*n* = 54) and diphenhydramine (*n* = 12). When specified, dosages, route(s) of administration, toxicity symptoms (including both physical and psychiatric ones), and outcomes were here reported.

**Conclusion:** Results from the systematic review showed that the OTC misusing issues are both widespread worldwide and popular; vulnerable categories include adolescents and young adults, although real prevalence figures remain unknown, due to a lack of appropriate monitoring systems. Considering the potential, and at times serious, adverse effects associated with OTC misusing issues, healthcare professionals should be vigilant, and *ad hoc* preventative actions should be designed and implemented.

## Introduction

Since generally being considered safe, over-the-counter (OTC) medicines are available without a prescription and can be purchased directly from related pharmacies/stores (1, 2). OTC medicines are meant to treat a variety of illnesses and symptoms, including pain, coughs and colds, diarrhea, nausea, etc. OTC availability, while encouraging self-care, has contributed to a public perception of safety and a lack of awareness relating to their potential for misuse, dependence, and harm (3–6). Indeed, some OTC medicines have active ingredients possessing a misusing potential at higher-than-recommended dosages (7) and are becoming increasingly popular for the possibility of their diversion in order to reach central psychoactive effects (8–11). Currently, there is minimal information about the prevalence of OTC misuse, abuse, and dependence (8–10, 12). Indeed, current lack of knowledge may partly be due to poor sales' monitoring because of OTCs' favorable legal status. However, the so-called “pharming” phenomenon (13–15) has been requiring attention at different levels because of increased treatment admissions, dangerous behavior, more emergency room visits, drug-related deaths, and overdoses (11, 16, 17). Most implicated drugs include certain cough suppressants, sleep aids, and antihistamines, which can at times be ingested in combination with remaining recreational psychotropics and/or prescription drugs and/or alcohol (17, 18). Overall, the misuse of OTC drugs is considered as more socially acceptable, less stigmatizing, and safer than the intake of illicit substances, also due to their likely lack of detection in standard drug screens (16). OTC drugs' intake may involve snorting or injecting the crushed tablets' powder to amplify the effects of a drug or ingesting these molecules for a purpose different from the therapeutic one. This may be the case for dextromethorphan (DXM) and codeine-based cough mixtures, being possibly misused at high dosages for recreational or euphoric effects; conversely, loperamide is at times being ingested for self-medicating withdrawal symptoms (7, 16, 18–20). OTC misuse has also been associated with notable drug interactions, physical and mental health effects, individual variation in responses, and significant socioeconomic impact for the users, their family, and the wider community (13–15). Currently, most OTC misusing data are obtained through clinical records (e.g., case reports and case series) and surveys.

### Aims of the Study

Thus, the current review aimed at (i) examining the current literature on the misuse of OTC drugs, focusing on the following OTCs: among antihistamines, diphenhydramine (DPH), promethazine, chlorpheniramine, and dimenhydrinate (DH); DXM- and codeine-based cough medicines; and the nasal decongestant pseudoephedrine; (ii) illustrating patterns of OTCs' misuse, psychopathological effects, and harms associated; and (iii) better understanding the psychotropic molecular mechanisms underlying their recreational use.

## Methods

### Systematic Review Procedures

A systematic electronic search was conducted from October 2020 to December 2020 and was set without a timeframe on the following scientific search engines: PubMed, Scopus, and Web of Science (WoS). The gray literature was also checked for relevant information. The following search strategies were used, respectively, in PubMed: (“diphenhydramine” OR “promethazine” OR “chlorpheniramine” OR “dimenhydrinate” OR “dextromethorphan” OR “pseudoephedrine” OR codeine-based cough medicines) AND (“abuse” OR “misuse” OR “craving” OR “addiction”) NOT review NOT (animal OR rat OR mouse) NOT “*in vitro*;” in Scopus: [TITLE-ABS-KEY (“Diphenhydramine”) OR TITLE-ABS-KEY (“Promethazine”) OR TITLE-ABS-KEY (“Chlorpheniramine“) OR TITLE-ABS-KEY (“Dimenhydrinate”) OR TITLE-ABS-KEY (“Dextromethorphan”) OR TITLE-ABS-KEY (“Pseudoephedrine”) OR TITLE-ABS-KEY (codeine-based cough medicines) AND TITLE-ABS-KEY (“Abuse”) OR TITLE-ABS-KEY (“Misuse”) OR TITLE-ABS-KEY (“Craving”) OR TITLE-ABS-KEY (“Addiction”) AND NOT TITLE-ABS-KEY (Review) AND NOT TITLE-ABS-KEY (animal) OR TITLE-ABS-KEY (rat) OR TITLE-ABS-KEY (mouse) AND NOT TITLE-ABS-KEY (“*in vitro*”)]; and WoS: (“diphenhydramine” OR “promethazine” OR “chlorpheniramine” OR “dimenhydrinate” OR “dextromethorphan” OR “pseudoephedrine” OR codeine-based cough medicines) AND (“abuse” OR “misuse” OR “craving” OR “addiction”) NOT Review NOT (animal OR rat OR mouse) NOT “*in vitro*.” The systematic review was structured in accordance with the Preferred Reporting Items for Systematic Reviews and Meta-analyses (PRISMA) (21) and PROSPERO guidelines (22). All data collected were tabulated on an Excel sheet to enable easy comparison and analysis.

### Data Synthesis Strategy

The selection and eligibility phase of the articles was carried out by three independent reviewers (AM, AMo, and MCS), who screened articles based on title and abstract; the first screening was followed by full text reviews, using predetermined criteria for inclusion and exclusion. Eligible articles were considered if the published studies met all the following criteria: (i) original articles (open-label or double-blind trials, prospective or retrospective observational studies, case series and case reports); (ii) studies involving all age individuals misusing the OTC drugs selected. There were no other restrictions on the type of study population or publication time period. Exclusion criteria were as follows: (i) nonoriginal research articles (e.g., review, letter, commentary, editorial, book chapter, professional or clients' opinions); (ii) non full-text articles (e.g., meeting/conference abstracts); (iii) languages other than English; (iv) animal/*in vitro* studies; (v) articles mentioning OTC drugs only as an example in the context of OTC drugs misuse; and (vi) articles not dealing with the misuse of the OTC drugs selected (e.g., DPH, promethazine, chlorpheniramine, and DH; DXM- and codeine-based cough medicines; and pseudoephedrine). Individual studies were also manually searched to identify additional citations. A final, between reviewers, cross-check was carried out, supervised by SC and MP, with both doubtful cases and possible inclusion/exclusion disagreements resolved through discussion with GM, MDG, and FS.

### Protocol and Registration

Current research methods were approved by PROSPERO (identification code CRD42020209261).

### Risk of Bias

The assessment of risk of bias was made in accordance with the Cochrane risk of bias 2 (RoB 2) tool (23).

## Results

**Figure 1:**
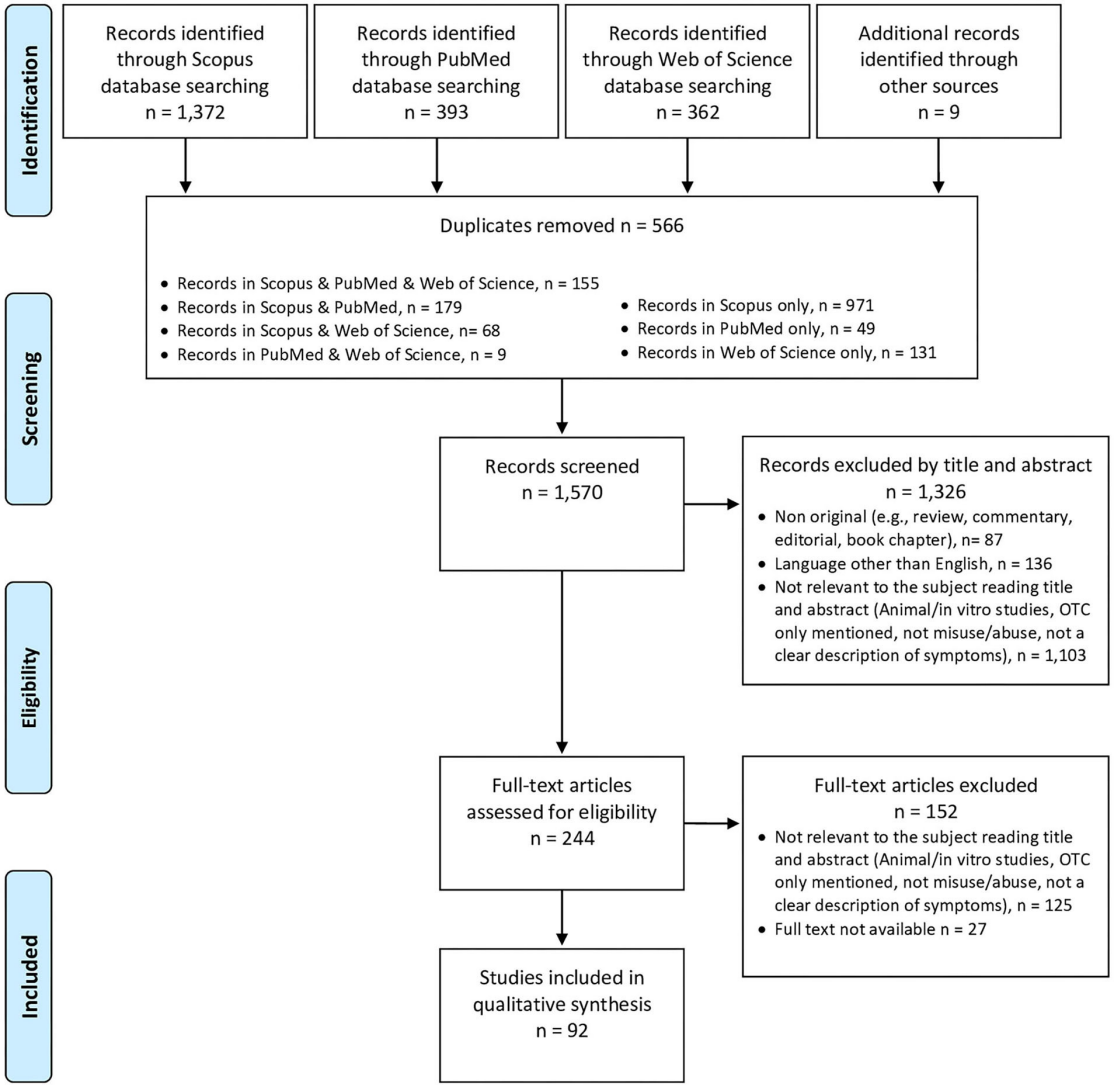
PRISMA flow diagram.

In removing duplicate articles (*n* = 566) from a total of 2,136 papers (PubMed = 393; Scopus = 1,372; WoS = 362; additional sources = 9), some 15,70 records resulted to be relevant for screening. Those considered not relevant to the subject while considering both the title and the abstract (*n* = 1,103; e.g., animal/*in vitro* studies; articles only mentioning OTC drugs, or not regarding OTC misuse/abuse, or not giving a clear description of related symptoms), those not written in English (*n* = 136), and those that were non-original articles (*n* = 87) were eliminated. Out of the 244 remaining full-text articles assessed for eligibility, some 125 papers did not match the inclusion criteria and 27 were not available. Hence, 92 articles were taken into consideration and properly analyzed (Figure 1). Findings were organized according to the specific OTC recorded, reported in alphabetical order in Supplementary Table 1; conversely, the most relevant characteristics of the misusing potential of the range of OTC drugs commented are summarized in Table 1.

**Table 1:** Drug classification and main characteristics of misuse of the selected OTC drugs.

**Drug/drug classification**	**Administration path**	**Mechanism of action**	**Effects**	**Does it cause dependence?**	**Street names and brand names**
Chlorpheniramine (antihistamine)	Oral	• Chlorpheniramine acts primarily as a potent H1 antihistamine drug • Moderate anticholinergic activity • Chlorpheniramine has been found to act as a serotonin reuptake inhibitor	• ACUTE EFFECTS: *psychiatric effects:* (i) sedating and anxiolytic properties; (ii) its abuse has been related to pleasurable feelings such as euphoria and stimulating effects; (iii) it may be associated with psychotic symptoms in predisposed individuals (e.g., people with mental illnesses or individuals concomitantly abusing other drugs) • CHRONIC EFFECTS: dependence	• Drug dependence is recorded after long-term use • Withdrawal symptoms, including excessive irritability, anger outbursts, insomnia, sweating, and craving	“Triple c” refers to Coricidin® cough and cold tablets; the combination of codeine, methyl ephedrine chlorpheniramine, and caffeine is marketed as Bron® Panadol® is a combination of chlorpheniramine, paracetamol and pseudoephedrine; Advil® includes ibuprofen, chlorpheniramine and phenylephrine; other brand names: Polaramine®, Chlortrimeton®
Codeine (opioid)	Oral, IV	• It is a selective agonist of the mu-opioid receptor; it is a natural isomer of methylated morphine, requiring metabolic activation by O-demethylation to morphine by CYP2D6	• ACUTE EFFECTS: *psychiatric effects:* euphoria, elation, analgesia, calmness; *physical effects:* respiratory depression, extreme somnolence progressing to stupor or coma, skeletal muscle flaccidity, cold and clammy skin, and sometimes bradycardia and hypotension. The triad of coma, pinpoint pupils, and respiratory depression is strongly suggestive of opiate poisoning. In severe overdosage, death may occur • CHRONIC EFFECTS: dependence	• Codeine has an identified abuse liability potential, given its effect and development of tolerance within a short timeframe on regular or excessive use • Codeine-dependence was here recorded, and associated with daily use of codeine	Street names: “Captain Cody,” “Cody,” “Little C,” “Schoolboy,” “Doors & Fours.” Common brand names for codeine and codeine containing combinations: Aspalgin® for aspirin and codeine; Nurofen Plus® for ibuprofen and codeine; Panadeine Forte® for paracetamol and codeine
Dextromethorphan (DXM) (non-competitive NMDA receptor antagonist and sigma 1 agonist antitussive)	Oral; IV and IN use also recorded in misuse cases	• At high doses, acting as a NMDA receptor antagonist, DXM and its potent metabolite dextrorphan inhibit the excitatory amino acid and neurotransmitter glutamate, causing hallucinogenic and dissociative states • DXM also exhibits binding activity at serotonergic receptors	• Neurobehavioural effects begin within 30–60 min of ingestion and persist for approximately 6 h • They are dose-related, starting from a mild to moderate stimulation with restlessness and euphoria (100–200 mg), to a state characterized by hallucinations, paranoia, perceptual distortions, delusional beliefs, ataxia, and out-of-body experiences (>1,000 mg) • ACUTE EFFECTS: (i) *psychiatric effects*: euphoria, altered mental status, mania, mood lability, irritability, dysphoria, insomnia; (ii) *physical effects*: tachycardia, hypertension, vomiting, mydriasis, diaphoresis, nystagmus, dystonia, loss of motor coordination; • CHRONIC EFFECTS: (i) toxic psychosis and cognitive deterioration; (ii) folate deficiency and neuropathy; (iii) since DXM is produced as the crystalline hydrobromide salt, bromism is a rare consequence that has been identified in heavy chronic abusers of DXM (neurotoxic effects, resulting in somnolence, psychosis, seizures, and delirium	• Although DXM is not thought to have addictive properties, its chronic use might determine addiction due to GABAergic/antiglutamatergic mechanisms, including substance-taking compulsive behaviors, tolerance, and autonomic withdrawal symptoms • EMCDDA: regarded as NPS	Street names: “Bromage,” “Brome,” “Candy,” “Dex,” “Dextro,” “DM,” “Drex,” “DXM,” “Red Devils,” “Robo,” “Rojo,” “Skittles,” “Triple C,” “Tussin,” “Velvet,” and “Vitamin D,” “Poor Man's Ecstasy”; the practice of using large amounts of DXM to achieve psychoactive effects is known as “robotrippin.” Common brand names are: Balminil DM®, Benylin DM®, Bronchophan®, Buckleys D®, Calylin #1, Delsym®, Koffex DM®, Novahistex DM®, Robitussin®
Diphenhydramine (DPH) (antihistamine moiety of dimenhydrinate/DH)	Oral; IV and IN use also recorded in misuse cases	• It is a first generation H1-antihistamine • Diphenhydramine also acts as a potent anticholinergic agent • It can acutely block the cell membrane pump mechanism of central 5-hydroxytryptophane and peripheral noradrenaline neurons	• ACUTE EFFECTS: (i) *psychiatric effects*: euphoria, altered mental status, hallucinations, and/or psychosis; (ii) *physical effects*: tachycardia, xerostomia, mydriasis, blurred vision, ileus, urinary retention, CNS depression, agitation, and hyperactivity • CHRONIC EFFECTS: dependence	• Reported cases of DPH dependence have resulted from usage of large doses (often over 1,000 mg per day) over periods of months or years. Withdrawal symptoms include craving, worsening of insomnia, rhinorrhoea, nausea, irritability, restlessness, abdominal cramps, sweating, and diarrhea. Gradual tapering has been the only described detoxification treatment plan	Different brand names, including Benadryl®, Dimedrol®, Daedalon®, Sominex®, Unisom® and Nytol®
Promethazine (antihistamine)	Oral	• It is a phenothiazine derivative and a H1 receptor antagonist; It also acts as a direct antagonist at muscarinic (M1) and dopamine (D2) receptors. It is classified as a first-generation antihistamine molecule which easily penetrates the blood-brain barrier and is associated with adverse effects such as sedation	• ACUTE EFFECTS: from mild sedation and CNS depression to profound hypotension, respiratory depression, unconsciousness, and sudden death; overdosage might determine an antimuscarinic delirium, agitation and neuroleptic malignant syndrome • it can be used to enhance effects of other co-ingested substances, e.g., opioids • CHRONIC EFFECTS: NR	• EMCDDA: regarded as NPS • Dependence might develop after long-term use of promethazine cough mixtures (containing opioids)	Promethazine mixed with a soft drink and/or alcohol is known as “purple drank,” “lean,” “syzzurp,” “Texas tea”; Phenergan® and Phenadoz® are common brand names
Pseudoephedrine (decongestant)	Oral; IV use also recorded in misuse cases	• Sympathomimetic properties, exerting a stimulating action on alpha, beta1-, and beta2-adrenergic receptors	• ACUTE EFFECTS: stimulant effects, e.g., euphoria, insomnia, diminished sense of fatigue, anorexia, and accelerated thinking; psychotic symptoms with auditory and visual hallucinations, persecutory delusions, fear, disorganized behavior might develop after high-dose consumption • CHRONIC EFFECTS: dependence	• Dependence might be developed after long-term use • Withdrawal symptoms include: dysphoria, restlessness, abnormal perceptions • Due to the possibility to be used to manufacture the class A controlled drug methylamphetamine, restrictions have been in place in the UK to manage the risk of products containing pseudoephedrine and ephedrine; in the US, a prescription is not needed in most States, and in remaining States there are limits on how much an adult subject can buy each month	“Chalk,” “Crank,” “Meth,” “Speed”; ‘Russian Cocktail' includes pseudoephedrine consumed together with potassium permanganate and acetylsalicylic acid diluted in water; common brand names: Sudafed®, Nexafed®, Zephrex-D® Claritin® includes pseudoephedrine and loratadine

*CNS, central nervous system; DH, Dimenhydrinate; DPH, Diphenhydramine; EMCDDA, European Monitoring Centre for Drugs and Drug Addiction; GABA, Gamma-Amino-Butyric Acid; H, Histamine; IN, Intranasal; IV, Intravenous; NMDA, N-Methyl-D-Aspartate; NPS, New Psychoactive Substance; OTC, Over-The-Counter; 5-HT, Serotonin*.

### Dextrometorphan

DXM resulted to be the most reported misused drug, with *n* = 54 related papers having been here identified (Supplementary Table 1). Indeed, it was recorded in two retrospective studies (24, 25), in 10 case series (26–35), and in several case reports (24, 25, 36–77). Most represented users were male adolescent and young adults; DXM was mostly used alone (28, 36, 37, 40, 44, 45, 54, 57, 66) or in DXM-containing cough mixtures (26, 29, 30, 39, 41, 42, 47, 50, 52, 53, 62, 64, 68, 71, 72, 74, 76). Concomitant drugs included both licit and illicit substances, such as alcohol (25, 30, 31, 35, 52, 53, 55, 60, 71, 76); cannabis (25, 31, 35, 48, 60); sedatives drugs, e.g., benzodiazepines (35); diethylamide lysergic acid (LSD) (35); opioids, e.g., morphine, heroin (25, 35, 54); ecstasy (35); cocaine (35); and phencyclidine/ketamine (34, 35). Dosages varied among cases, up to super-high dosages (up to 4,920 mg) (31, 35, 36, 61). The only route of administration (ROA) here recorded was the oral one. Autonomic (e.g., mydriasis, tachycardia, palpitations) (30, 33, 35, 42, 44, 46, 47, 51, 67, 70, 71), gastrointestinal (32, 35, 42, 47), neurological [e.g., amnesia, nystagmus, ataxia, seizures, and dystonia; (24, 26, 29, 30, 32, 34, 35, 39, 43–46, 49, 51–53, 56, 59, 67)], and psychiatric symptoms, such as euphoria, agitation/irritability, confusion, hallucinations, and delusions, have been recorded (24, 25, 27–31, 33–38, 40–50, 52–54, 56, 58, 60, 61, 63, 66, 67, 70–74, 76). DXM misusers' psychiatric history frequently included alcohol and substance use disorders (SUD) (25–27, 29, 31, 32, 34–37, 40, 43, 45–48, 50, 53, 55–62, 64–67, 69, 76), mood disorders (29, 31, 32, 35, 37, 38, 41, 46, 56–65, 67, 68, 71), and schizophrenia (37, 53, 69). Regarding the outcome, most cases required hospitalization with supportive treatments and antipsychotics [e.g., haloperidol (43, 47, 71, 73, 75)], risperidone (74), and olanzapine (54, 61) administration. A DXM-related suicide has been recorded (31).

### Chlorpheniramine and Codeine

Chlorpheniramine and codeine were recorded as having been misused in two papers (respectively, 68 and 69), as constituents of BRON, a Japanese codeine-based cough suppressant, together with methyl-ephedrine and caffeine (78, 79). BRON abuse has been associated with both psychotic/affective symptoms and dependence/withdrawal issues (78). Moreover, a case of severe intoxication of a codeine-based cough mixture determining a respiratory acidosis and requiring hospitalization was recorded (80) (Supplementary Table 1).

### Dimenhydrinate

DH misuse was described in eight articles (Supplementary Table 1), including five case reports (81–85) and three case series (86–88), mostly involving adults or adolescents (88). Most important psychiatric comorbidities described were represented by mood disorders (82, 84), SUD (83–87), and schizophrenia (85, 87). Massive dosages, up to 5,000 mg, of DH have been recorded in a few cases (84, 85, 87). DH administration was always oral, except for one case where the molecule was administered intramuscularly in association with opiates and benzodiazepines (83). The symptoms recorded ranged from recreational stimulating effects (87) to emotional lability, agitation, anxiety, and drug-induced delirium with paranoia, thought incoherence, and visual/auditory hallucinations (81, 86). The physical effects reported were mild and included mydriasis, tachycardia, hypertension, flushing, restlessness, dystonic reactions, and ataxia (81, 82, 84–86, 88), while one case reported generalized seizures (87). Withdrawal symptoms have been recorded after the abrupt interruption of chronic use and included irritability, anxiety, and craving (82, 84, 87). When reported, treatment was almost supportive (81–83, 85, 88); in two cases, benztropine was required to treat dyskinesia and related movement, muscle control, and balance symptoms (81, 84).

### Diphenhydramine

DPH misuse was reported in 12 articles, including 10 case reports (17, 89–97); the remaining two included, respectively, a case series (98) and a retrospective review study (99) (Supplementary Table 1). Apart from the retrospective review study focusing on all Mandrax® (DPH + Methaqualone) abuse cases (*n* = 67, male) retrieved from the United States (US) Army during January–June 1972, users were here mostly represented by female (F/M, 9/6). A high number of users were adolescents, aged between 13 and 18 years (17, 94, 96–98). Reported psychiatric comorbidities mostly included SUD (17, 89–92, 95), schizophrenia/psychotic symptoms (89, 91, 92), and mood disorders (17, 90, 91). DPH was taken in most cases orally, but both intramuscular (IM) (90) and intravenous (IV) (96–98) administrations were reported as well. Super-high dosages were recorded, up to 2,000 mg daily (91–93, 98). In a few cases, DPH was misused together with alcohol (91, 99), lorazepam (98), and cannabis (99). A polydrug overdose (e.g., DH together with bupropion, citalopram, acetaminophen, omeprazole, and naproxen) was recorded (94). DPH recreational use was associated with relaxation, calmness, and sleep improvement (90, 92, 96, 98, 99). Acute intoxication was associated with psychotic symptoms, psychomotor agitation, restlessness, and disorientation (89, 92–96, 98, 99). Withdrawal, consisting in both physical (e.g., bowel and bladder incontinence, hypertension, hypertonia, and extrapyramidal symptoms) and psychological (e.g., anxiety, irritability, rebound insomnia, and craving) symptoms have been recorded (17, 89, 90, 92, 95, 98, 99). DPH-induced intoxication was associated with signs and symptoms of anticholinergic toxicity, such as fever, mydriasis, flushed skin, dry mouth, dry eyes, decreased sweating, urinary retention, and dyskinesia (92–94, 98). A severe toxicity case was associated with cardiac conduction abnormalities and increased QT interval (90). On-drug cases of violent behavior, including suicide, have been reported (97, 99). Treatment required hospitalization and supportive care; drugs used were antipsychotics, such as fluphenazine and quetiapine, benzodiazepines, and benztropine (89, 90, 92, 93).

### Promethazine

A few papers recorded here the misuse of promethazine; a retrospective analysis of data from the American Association of Poison Control Centres (AAPCC) National Poison Data System (NPDS) from January 2002 to December 2012 reported 354 promethazine intentional misuse/abuse cases (100) (Supplementary Table 1). All cases involved adolescents and young adults who misused promethazine orally. In most cases (*n* = 259) promethazine abuse was associated with other substances, such as DXM, codeine, phenylephrine, pseudoephedrine, caffeine, etc. Intoxication symptoms ranged from mild to severe effects, up to seizures and coma, but no fatalities have been reported. Agitation, confusion, slurred speech, and hallucinations were described as well. Promethazine-alone abuse cases were mostly managed in healthcare facilities, while promethazine in coformulation mostly required emergency department (ED) care management (100). Moreover, further cases of nonmedical use of promethazine were here identified from (i) the Danish Poison and Information Centre (DPIC) and related registers used within the State Serum Institute of Denmark (SSI) (101); (ii) a prospective database of poisoning admissions (January 1987-May 2007) to a UK regional toxicology service (102); and (iii) a prospective study regarding patterns of misuse of heroin injectors (103). Drug-induced delirium was the most represented psychiatric effects; this was managed with antipsychotics and benzodiazepines (101, 102). Interestingly, the use of promethazine injection in opioid users was reported as a substitute for heroin or to increase the effects of an inadequate heroin dosing (103). A case of drug-induced delirium deriving from the coingestion of high-dose promethazine, cyproheptadine, and fluvoxamine in a young girl was recorded (104). Finally, a case of promethazine dependence and withdrawal after 2-year continuing use of a promethazine–cough mixture was described (105).

### Pseudoephedrine

Seven articles, including six case reports (106–111) and one case series (112), described the misuse of pseudoephedrine (Supplementary Table 1). Cases mostly involved male adults (age range, 18–45 years) (F/M, 3/7) suffering from mood disorders (107, 109–111). One paper recorded an SUD [e.g., alcohol, cannabis, and heroin; (112)]. Massive dosages [e.g., 3,000–4,500 mg of pseudoephedrine/day; (107)] and IV administrations (108, 111, 112) have been associated with the misuse of pseudoephedrine. Physical symptoms associated with pseudoephedrine high dosage ingestion included stimulating effects such as decreased appetite, dry mouth, palpitations (106, 107, 112), and motor symptoms [e.g., gait and balance disorder, postural instability, generalized dystonia, hypokinesia, bradykinesia, psychomotor retardation; (106–108, 112)]. Pseudoephedrine effects were dose dependent and ranged from euphoria, insomnia, diminished sense of fatigue, and accelerated thinking, to psychotic symptoms with auditory and visual hallucinations, persecutory delusions, fear, and disorganized behavior (106, 109–111). Withdrawal symptoms have been recorded after the abrupt interruption of the long-term use (106, 107). Some cases required hospitalization and treatment with antipsychotics, e.g., haloperidol (106, 109–111); benzodiazepines (108); and antidepressants, e.g., amitriptyline (106, 108). No fatalities have been recorded.

## Discussion

This systematic review has illustrated a range of both themes and data regarding the misuse/abuse of some selected OTC drugs, including DXM, DPH, DH, codeine-based cough syrups, promethazine, and pseudoephedrine. Their misuse potential may be particularly significant in adolescents and young adults (10, 12, 113). OTC recreational intake appeared to be associated with high/very high dosages (17, 27, 30, 31, 35, 36, 40, 42, 45, 46, 55, 58, 61, 66, 76, 79, 84, 85, 88, 90–93, 104, 107, 114); idiosyncratic routes of administration (e.g., snorting; IM; IV; 39, 69, 88–90, 100, 103); and associated with ingestion of both licit [e.g., alcohol, prescription opioids, benzodiazepines, other OTCs; (25, 35, 49, 52–55, 60, 61, 72, 76, 83, 91, 94, 99, 101, 102)] and illicit (e.g., cannabis, cocaine, ketamine, etc.) drugs (30, 31, 34, 35, 48, 58, 60, 61, 88, 99). OTC drugs were obtained by various means (8–11), including family and friends (63), multiple doctor prescriptions (27, 36, 63, 90, 93), illegal online pharmacies/shops (36, 42, 70, 77), and theft/burglary from hospitals, residences, and pharmacies (27, 105, 110). DXM pills named “Snurf” were also reported to have been acquired online and in having been marketed as a legal high (70).

Overall, two main populations of OTC misusers were identified (11): (a) patients already suffering from a health condition and/or a psychiatric disorder who became dependent on their prescription/OTC drugs due to prolonged/high-dosage use (115), e.g., DXM-based cough mixtures started for sinusitis, cough, nasal congestion, and then continued for years at higher dosages (27, 58). Other examples have included DH prescribed for emesis in pregnancy and then continued for 12 years at a higher dosage without a prescription (82), DPH use initiated to assist with initial insomnia and then continued for 6 months up to 1,600 mg daily (92), and pseudoephedrine self-administered to lose weight then causing addiction (106); (b) individuals, including substance abusers, not in treatment for a medical disorder or illness who may have started to misuse/abuse with OTC medications for recreational purposes (36, 40, 43, 45, 70, 116).

Out of a total of *n* = 185 OTC misusers described in case reports/series surveys (24, 25, 77, 78, 99–103), male subjects were the most represented (F/M = 51/134), with an SUD history having been recorded in 53 of them (53/185 = 28.6%). A range of psychiatric diagnoses were reported (45/185 misusers, 24.3%), including mood disorders (e.g., bipolar disorder, depression, dysthymia; *N* = 26), anxiety disorders (e.g., adjustment disorder, anxiety; *N* = 5), psychotic disorders (e.g., schizoaffective disorder, schizophrenia, psychosis, delusional disorder; *N* = 11), attention deficit and hyperactivity disorder (ADHD, *N* = 1), eating disorders (e.g., bulimia; N = 1), and personality disorders (e.g., dependent disorder; *N* = 1). Regarding the outcome, most cases recorded were associated with a full recovery after hospitalization, with treatment having been either supportive (32, 44–46, 65) or symptomatic, with the latter consisting of benzodiazepines and antipsychotics (25, 27, 28, 43, 47, 49, 51, 54, 61, 67, 68, 71, 73–75, 79, 111, 115). A full detoxification procedure was recorded in cases of dependence and withdrawal (17, 82, 92, 95, 98, 105, 107, 109, 115); examples included buprenorphine 2 mg/day to treat a sudden opiate (codeine) withdrawal symptoms (114), naltrexone as a relapse prevention agent for DXM dependence (63), and topiramate for DXM craving (56). Some cases required specific actions in the Emergency Unit (80). Finally, it has been suggested here that drug use treatment would benefit from counseling, behavioral therapies support, and rehabilitation treatment to better overcome drug craving (11, 18, 27, 28, 34, 36, 46, 48, 53, 59, 60, 78, 84, 110, 117). OTC-related fatalities were here related to either cases characterized by unusually high dosages (24, 31, 96) or to suicide/self-aggression (31).

The cough-suppressant DXM resulted here to be the most popular OTC being misused (Supplementary Table 1) due to its dose-dependent sedative, dissociative, and stimulant properties (16, 118–120). Indeed, DXM psychotropic effects are mostly related to its active metabolite dextrorphan, which, if used in large dosages, is able to antagonizes N-methyl-D-aspartate (NMDA) receptors, hence modulating the excitatory neurotransmission; this results in the production of specific dissociative, ketamine-like, experiences (19, 25, 31, 56, 118–121) (Supplementary Table 1 and Table 1). The effects depend upon several factors, such as an individual's CYP2D6 subtype, body weight, as well as the degree of tolerance to DXM, and the concomitant use of other CYP2D6 substrates, including antidepressants (fluoxetine, fluvoxamine, nefazodone, paroxetine, sertraline, venlafaxine), antipsychotics (clozapine, haloperidol, risperidone, thioridazine), β-blockers (atenolol, metoprolol, propranolol), antiarrhythmics, and opioid analgesics (codeine, tramadol, and methadone), which may decrease the rate of DXM metabolism, resulting in a DXM intoxication (13, 19, 47, 121, 122). Due to DXM catabolism by repeated demethylation, which may lead to abnormal folate demands for methyl group transfer, a folate deficiency has been described in association with chronic DXM use (26, 39, 122). In addition, dental caries cases were associated with the high syrup content of cough mixtures (26). Although DXM is not thought to have addictive properties, with chronic use, vulnerable individuals may rapidly develop tolerance, dependence, and withdrawal (35, 36, 56, 58, 63, 66, 76). Interactions with other substances can often produce synergistic effects; in fact, OTC cough formulations frequently contain, in addition to DXM, other pharmaceutical agents such as chlorpheniramine, acetaminophen, or pseudoephedrine, exhibiting different effects. Indeed, individuals abusing with chlorpheniramine-containing DXM formulations may also exhibit anticholinergic signs and symptoms (25, 31, 42, 47, 49, 73, 74, 123). Conversely, the antipyretic and analgesic acetaminophen produces delayed hepatic injury (29, 62). Finally, interactions between DXM and selective serotonin reuptake inhibitors (SSRIs) or monoamine oxidase inhibitor (MAOI) might further increase the risk of a serotoninergic syndrome occurrence (67, 68, 121, 124).

Although widely used and generally considered safe, cases of antihistamine abuse and dependence have been recorded (125). These molecules were originally marketed for their antiallergy properties and are now made available as sleeping aids. Antihistamines' toxicity appears to be clinically related to both central and peripheral acetylcholine antagonism. In addition, specifically due to multiple potential mechanisms of action, DPH (e.g., the antihistamine moiety of DH) can acutely block the cell membrane pump mechanism of central 5-hydroxytryptophane and peripheral noradrenaline neurons, causing the euphoria reported by some users (Table 1). At high dosages, and taken together with other drugs (e.g., alcohol, cannabis, and stimulants), DPH and DH might be used to achieve a stimulant effect (87, 91, 92, 126, 127). Reported cases of DPH dependence have resulted from long-term usage of large doses (often over 1,000 mg/day). Gradual tapering has been described to alleviate withdrawal symptoms (17, 125). Conversely, promethazine is used in cough syrups for its antihistaminic, antiemetic, and sedative effects, available with codeine in common cough suppressants (128); its abuse potential appears related to its calming and sedating effect and enhancement of other coingested substances (Table 1). A recreational use of promethazine mixed with a soft drink and/or alcohol (“purple drank”) is currently popular among young people for its euphoric effects and easy accessibility (19, 20, 129–131). Promethazine has been reported in SUD clients and is misused as a substitute for another drug or to increase the effects of inadequate dosing (i.e., to delay the onset of opioid withdrawal or to potentiate the sedating effect of benzodiazepines/Z-drugs) (13, 19, 20, 103, 129, 130, 132, 133). Overdose of promethazine is associated with an antimuscarinic delirium, agitation, and neuroleptic malignant syndrome (100, 102, 104, 133). Scott et al. (104) recorded a promethazine-induced delirium treated with physostigmine intravenously, which reversed both central and peripheral anticholinergic effects, similarly to a polydrug overdose due to the ingestion of DPH (94). Chlorpheniramine is used as a cheap sleep aid and/or as an anxiolytic due to its antimuscarinic properties; its abuse has been related to pleasurable feelings, which reinforces the repetitive use and the possibility of developing drug dependence (Table 1). It may, however, be associated with psychotic symptoms in predisposed individuals [e.g., people with mental illnesses or individuals concomitantly abusing other drugs; (42, 43, 114, 115)].

Codeine was reported within the misusing scenario of codeine-based cough and cold medicines and/or coingested with other substances, e.g., DXM, DPH, ephedrine, pseudoephedrine, methyl ephedrine, chlorpheniramine, promethazine, caffeine (26, 27, 34, 78–80, 100, 114, 134). Codeine is a natural isomer of methylated morphine and, similarly to DXM, is a prodrug, requiring metabolic activation by O-demethylation to morphine by CYP2D6. Thus, codeine-related effects are associated with CYP2D6 metabolism, e.g., ultrarapid CYP2D6 metabolizers produce an unexpectedly large amount of morphine, with resulting life-threatening opioid toxicity. Its recreational use is related to the agonism at mu receptors and the subjective effects of euphoria, elation, analgesia, and “liking” (114, 121). Codeine toxicity is characterized by respiratory depression and extreme somnolence progressing to stupor or coma (79); in severe overdosage cases, death may occur (121) (Supplementary Table 1 and Table 1). Idiosyncratic codeine administration procedures have been recorded, e.g., a misuser learned online how the codeine base might be extracted through a process called cold water extraction (CWE) to be then injected. Regular use of codeine is described here together with the development of both tolerance (135) and dependence (80, 114).

Decongestants, here recorded as being abused, both alone and with coingestants, were ephedrine and its stereoisomer pseudoephedrine (78, 79, 106–109, 111, 112), which are sympathomimetic agents (136, 137) exerting a stimulating action on both alpha- and beta-adrenergic receptors (136, 137) (Supplementary Table 1 and Table 1). Indeed, ephedrine has been reported to obtain weight loss or to enhance athletic performance; both pseudoephedrine and ephedrine have been recorded as used illicitly in the production of methamphetamine (136, 138). The abuse was here associated with high dosage (106–109) and IV administration (108, 111, 112). Dependence issues have been recorded (106–109).

## Limitations

One of the difficulties regarding the literature on prescription drug misuse is both its heterogeneity and the issues in identifying misusing practices; interpretation was easier for both those cases reported by healthcare professionals, whose intervention was needed, National/Regional Poison Data System information (100, 101), etc. According to UNODC, the misuse of medicines is defined as “the problematic consumption outside of acceptable medical practice or medical guidelines, when self-medicating at higher doses and for longer than is advisable, for intoxicating purposes and when risks and adverse consequences outweigh the benefit” (8–11). However, levels of terminology variability and inconsistency to describe the OTC phenomenon were identified as well; this use was referred to as non-medical use, problem use, harmful use, recreational use, self-medication, or inappropriate use, which calls into question whether there is a consensus on the negative consequences (i.e., problem, harm) of OTC use. Indeed, some of these terms may not even necessarily refer to the same issue (8).

## Conclusions

The current systematic review showed that OTC misuse is an increasingly relevant health issue associated with potential harms, including drug-related toxicity, addiction, and fatalities. Nowadays, the CoViD-19 pandemic has likely facilitated the occurrence of these misusing practices, as more users turned from street drugs to prescription/OTC products (14, 15). Indeed, OTC drugs are both widely accessible and perceived because of their favorable legal status as relatively safe, hence accepted in a “pill-popping culture” (11). There is the need of both drafting *ad hoc* treatment guidelines and planning preventative measures. These measures should revolve around the implementation of a range of associated issues, including scheduling amendments, proper surveillance, enhanced detection of misuse in clinical and pharmacy practice, and promotion of public health awareness initiatives (9, 11, 16, 139–141). As an example, due to the recent rise in opioid abuse and related overdose deaths worldwide, efforts are focusing on strengthening public health surveillance and limiting opioid prescribing (142, 143). Specifically, as codeine-containing products misusing levels might be hampered by their widespread and easy availability, upscheduling and pharmacy-based interventions targeting users might limit the purchase of codeine products without a prescription. The recent introduction of new OTC combinations with non-opioid agents may provide a safer alternative to these widely misused products (144). In the case of the antidiarrheal loperamide, found to be misused at high dosages and associated with cardiotoxicity, to support its safe use, the Food and Drug Administration (FDA) approved changes to the packaging for tablet and capsule forms limiting each carton to no more than 48 mg of loperamide and requiring the tablets and capsules to be packaged in individual doses (145). A range of professionals should be involved in tackling the OTC misusing issues, including (i) physicians, especially general practitioners (GP), who can help OTC misusers in early recognizing a drug-related problem and refer them to the appropriate service (e.g., mental or addiction services); they should also take note of rapid increases in the amount of medication needed or frequent, unscheduled refill requests and uncovering possible “doctor shopping” practices. Physicians will continue to have a role in educating users to ensure that they use medications appropriately, following the prescribed directions, while being aware of potential interactions with other licit/illicit drugs (11, 16, 18, 116, 135, 141). Conversely, pharmacists should be watchful for prescription falsifications or alterations, being at the frontline in recognizing prescription drug abuse issues. Moreover, prescription drug monitoring programs could assist healthcare professionals in identifying patients who are getting prescriptions from multiple sources (11, 13, 16–18, 141, 144, 146). Finally, abuse prevention campaigns might provide valuable resources on raising awareness and preventing medicine abuse [https://stopmedicineabuse.org/; (144)].

## Data Availability Statement

The original contributions presented in the study are included in the article/Supplementary Material, further inquiries can be directed to the corresponding author/s.

## Author Contributions

FS, SC, and GM conceived the idea of this paper. AM, MCS, and AMo extracted the data. FS, MP, GM, AG, and MDG supervised all stages of the process and were consulted to resolve any possible disagreement. SC, AM, and JMC drafted the first version and revised it after contributions from FS, AG, and GM. All authors contributed to the article and approved the submitted version.

## Conflict of Interest

FS was a member of the UK Advisory Council on the Misuse of Drugs (ACMD; 2011–2019) and is currently a member of the EMA Advisory Board (Psychiatry). GM has been a consultant and/or a speaker and/or has received research grants from Angelini, Doc Generici, Janssen-Cilag, Lundbeck, Otsuka, Pfizer, Servier, Recordati. MDG has been a consultant and/or a speaker and/or has received research grants from Angelini, Janssen-Cilag, Lundbeck, Otsuka, Pfizer, Servier, Recordati. JMC is a member of the ACMD's Novel Psychoactive Substances and Technical Committees. The remaining authors declare that the research was conducted in the absence of any commercial or financial relationships that could be construed as a potential conflict of interest.
