# Supplementary material for: Focus on Over-the-Counter Drugs' Misuse: A Systematic Review on Antihistamines, Cough Medicines, and Decongestants
Source: Front Psychiatry. 2021 May 7;12:657397. doi: 10.3389/fpsyt.2021.657397 (PMC8138162; doi:10.3389/fpsyt.2021.657397)
Supplement: Supplementary file 1 [file Table_1.docx]

| Ref. | Study design | Population (N tot) | Mean age | Psychiatric comorbidity | Medical comorbidity | Dosage and ROA | Physical Symptoms | Psychiatric symptoms | Poly-abuse (substance) | Actions taken and outcome | Notes |
| --- | --- | --- | --- | --- | --- | --- | --- | --- | --- | --- | --- |
| Chlorpheniramine | | | | | | | | | | | |
| Das et al., 2017 | Case Report | N=1 (M) | Age= 34 yrs | Chlorpheniramine initiated as sleep aid, and then consumed at high dosage for 5 yrs | NR | 60mg/day, oral | Withdrawal symptoms, including excessive irritability, anger outbursts, insomnia, sweating, and craving | High dose misuse resulted in mood changes, euphoria, increased activity, anorexia, insomnia; delusion of grandeur, feelings of blissfulness, increased religiosity, and abnormal behaviour, e.g., wandering around in night | None | Admitted to the psychiatric department, diagnosed as bipolar disorder with current chlorpheniramine induced mania; detoxified by tapering the drug with valproate and olanzapine |  |
| Codeine and codeine-based cough mixtures | | | | | | | | | | | |
| Donuk et al., 2015 | Case report | N=1 (F) | Age= 17 yrs | None | None | Codeine content of 80-120 capsules, IV, SC | NR | Initial light sedation, followed by feelings of euphoria, muscular relaxation, and a general sense of well-being | None | Buprenorphine 2mg/day was given following sudden opiate withdrawal symptoms. Since early, she showed a persistent migraine-like frontotemporal headache, accompanied by photosensitivity, nausea, and vomiting, with both the neurological examination and the MRI unremarkable, and the ophthalmological examination reporting a bilateral papilledema; treated with suboxone and acetazolamide for the headache, which improved after 1 week. After 2 months, the patient was asymptomatic, with complete resolution of papilledema | From the Internet she learned how the codeine base might be extracted removing acetaminophen and aspirin through CWE process |
| Wong et al., 2001 | Case report | N=1 (M) | Age= 25 yrs | None | None | Toxicology screening showed metabolites of opiates, ephedrine/ pseudoephedrine, codeine, promethazine, and chlorpheniramine | Progressive generalized muscle weakness; bilateral symmetric limb weakness; deep tendon reflexes were diminished on both sides; hypokalemia associated with mixed normal anion gap hyperchloremic metabolic and respiratory acidosis | None | None | After admission potassium was replaced and the acidosis treated with sodium bicarbonate therapy | Metabolic acidosis was likely related to an overdose of ammonium, whereas respiratory acidosis (and hypoventilation) was probably related to the effect of hypokalemia (deriving from a transcellular shift of potassium induced by ephedrine/ pseudoephedrine) |
| Dextromethorphan (DXM) | | | | | | | | | | | |
| Akerman et al., 2010 | Case Report | N=1 (M) | Age= 17 yrs | ADHD, SUD (alcohol and cannabis) | None | He increased use up to 1,440 to 3,840 mg  per day, oral | NR | Well-being, calmness, weightlessness, increased concentration and focus. DXM and alcohol were associated with auditory and visual hallucinations, poor concentration, a feeling of displacement, cloudy thoughts, and physical stiffness. Over time, he developed dependence and eventually symptoms of withdrawal, including diaphoresis, flushing, tachycardia, weakness, nausea, insomnia, racing heart, anxiety, and craving | None | He was treated with clonidine, resulting in resolution of the vital sign abnormalities. He was discharged to an intensive outpatient program. Over several months, he maintained abstinence while participating in psychosocial treatment for addiction. Began taking atomoxetine for ADHD | Obtaining the medication from various pharmacies and also from Web sites on the Internet |
| Alam et al., 2013 | Case report | N=1 (M) | Age= 22 yrs | Cannabis-induced psychotic episode; Schizoaffective disorder (on treatment with antipsychotic) | NR | 600 mg daily, oral | NR | DXM use was firstly associated with euphoria and sense of dissociation; long-term usage determined psychotic symptoms (e.g., visual, and auditory hallucinations and paranoia), disorganized and intrusive behaviour, poor self-care, fatuous incongruous affect, and irritability, with no clear response to antipsychotic medication | None | After psychotic symptoms resolved, he maintained remission until he took DXM again |  |
| Amaladoss and Brien, 2011 | Case report | N=1 (F) | Age= 20 yrs | Adjustment disorder, insomnia | Pharyngitis and fibromyalgia. On treatment with oxycodone, acetaminophen, and clindamycin | DXM-containing cough syrup administered above the required and recommended dosage, oral | NR | Drug-induced psychotic symptoms, including bizarre paranoid and somatic delusions, visual and auditory hallucinations, disorganized and pressured speech, mood lability | None | After 4 days of hospitalization in the psychiatric unit, she improved with olanzapine and she was discharged |  |
| Au et al., 2003 | Case series | N=4 (M) | Mean age=28,25 yrs (SD=1,64) | Cases 1-4: NR | Cases 1-4: tobacco users | Case 1,2,4: chronic use of 500ml daily; case 3: chronic use of 1,000ml daily; oral | Peripheral neuropathy, dizziness, difficult walking, and macrocytic anaemia caused by folate deficiency | NR | Cases: 1-4: tox screening detected combinations of codeine, pseudoephedrine, DXM, promethazine, phenothiazine, diazepam, caffeine and chlorpheniramine | Treated with oral multivitamin and folate and IM vitamin B12, with complete neurological recovery and normalization of hemoglobin, mean corpuscular volume and vitamin B12 and folate levels at 3 months | Severe folate deficiency secondary to cough mixture abuse; dental carries associated with the high syrup content of cough mixtures |
| Au et al., 2005 | Case report | N=1 (M) | Age= 34 yrs | None | None | Cough DXM-mixture binges of 500ml  daily for two yrs, oral | Unsteady gait and clumsiness; sensory peripheral neuropathy; gross past pointing; dysdiadochokinesia; cerebellar gait and mild macrocytic anemia; teeth erosion; computerized tomogram scan showed bilateral cerebellar degeneration and thinning of folia | None | None | After three months of physiotherapy and vitamin supplements, there was recovery in gait and dexterity with residual cerebellar signs | . |
| Bernstein et al., 2019 | Case Report | N=1 (F) | Age= 37 yrs | Long-term misuse of DXM (5 yrs) | NR | 1,400mg of DXM over the course of 3 days, oral | NR | Psychotic and manic state with insomnia, euphoria, grandiose and religious delusions, auditory hallucinations influencing her behaviour (she used scissors attempting auto-enucleation) | None | She presented to the ED. She was medically cleared and after 3 days transferred to inpatient psychiatry for treatment of depression and discharged with citalopram | No other substances revealed by tox screenings |
| Bostwick, 1996 | Case report | N=1 (F) | Age= 35 yrs | Bipolar Disorder | NR | Several doses of Robitussin DM®,  Robitussin CF® and Vicks 44E® | NR | Substance-induced mania with insomnia; motor restlessness; racing and obsessive thoughts; increased energy and irritability | Phenylpropanolamine and guaifenesin | Symptoms resolved upon discontinuation of cough and cold medications | She was on treatment with Lithium |
| Boyer et al., 2004 | Case report | N=1 (F) | Age= 12 yrs | None | None | 16 gel tablets of Coricidin® (480mg of DXM), oral | Vomiting, mydriasis; marked lateral nystagmus; dry oral mucosa, and facial flushing | Euphoria, psychomotor agitation | Chlorpheniramine | In the ED treated with supportive care (IV fluids) during a brief observation period and was discharged after her vital signs normalized | Several DXM-containing products shoplifted from a local store |
| Butwicka et al., 2013 | Case report | N=1 (M) | Age= 16 yrs | History of misuse of DXM and pseudoephedrine | None | Unspecified dose, oral | Dystonia | Psychotic symptoms | None | Admitted to the pediatric neurology unit and treated with antipsychotics (olanzapine, levomepromazine, and haloperidol) developing a neuroleptic malignant syndrome, requiring ICU (intubation and mechanic ventilation). After 10 weeks of hospitalization, he was readmitted to the psychiatric unit and treated with gabapentin for seizures, and ziprasidone for psychotic symptoms. In June 2010 she was discharged diagnosed with schizophreniform disorder; he remained under regular review in the outpatient psychiatric clinic | Genotyping revealed that he was homozygous for a non-functional CYP2D6*4 allele, which might be a risk factor of adverse reactions, such as neuroleptic malignant syndrome |
| Cherkes and Friedman, 2006 | Case report | N=1 (M) | Age= 28 yrs | History of SUD (heroin, crack, alcohol) | None | Unspecified dose of DXM, oral | Light-headedness on standing, headache, sleepiness, psychomotor retardation, significant gait ataxia, nystagmus | Disorientation, reduced attention and interest in his environment, mild stupor | None | Discharged from the ED uneventfully after receiving supportive care | A urine specimen sent for a “comprehensive toxicology screen” was positive for DXM |
| Craig, 1992 | Case report | N=1 (F) | Age= 25 yrs | History of OTC drug abuse | None | 2,400mg of DXM, oral | Diaphoresis, horizontal nystagmus, hyperreflexia, tachycardia, hypertension | Tangential speech, labile affect, and paranoid psychosis with delusion; impaired attention and concentration | Phenylpropanolamine and guaifenesin | Discharged uneventfully after receiving supportive care |  |
| Desai et al., 2006 | Case report | N=1 (F) | Age= 66 yrs | Depression and SUD (alcohol) | Asthma, pulmonary embolism, upper gastrointestinal bleeding | 2 bottles of DXM-containing syrup (960mg of DXM daily) over an 8-year period, oral | Hypertension (183/91 mm Hg), tachycardia (96 bpm), hyperthermia, and respiration rate 16pm. The only findings noted on examination were a mild coarse tremor | Depression, dysphoria, insomnia | Urine toxicology screen positive for phencyclidine (false positive due to DXM) | Presented to the hospital ED, he was administered chlordiazepoxide to relieve symptoms of trembliness Discharged 2 days later with a treatment center referral, she was admitted to a detoxification program | On treatment with aspirin, salbuterol inhalers, fluticasone/ salmeterol inhaled powder, montelukast, pantoprazole, and supplemental calcium and vitamin D |
| Dilich and Girgis, 2017 | Case report | N=1 (M) | Age= 69 yrs | History of SUD (alcohol, opioid, and cocaine) in remission | History of seizures, hepatitis C, gastroesophageal reflux disease, hypertension, obesity, and degenerative joint disease | Many empty bottles of Robitussin  ® found lying around the house; estimated he was drinking several bottles/day | Tachycardia (100 bpm), hypertension (151/94 mmHg) | Intoxication delirium, including the following symptoms: psychomotor agitation; mood liability; disorganized thinking with racing thoughts and tangential thought process; insomnia; unusual and irregular behaviours; paranoid delusions; auditory hallucinations | On methadone maintenance program (150mg/day) | Brought to the ED by law enforcement for psychiatric evaluation after his family called the police. He became aggressive at staff and was medicated with haloperidol 5 mg and lorazepam 2 mg and subsequently placed in four-point soft restraints. Discharged after three days | On treatment with gabapentin, diclofenac, losartan, furosemide, and omeprazole |
| Fleming, 1986 | Case report | N=1 (M) | Age= 30 yrs | History of SUD (cannabis, amphetamines, and barbiturates) | None | Max 1gr of DXM powder, sniffed | Nausea | After consumption feeling high and restlessness; after two hours down with depressed mood, tiredness, and dizziness | Cannabis | Admitted to the drug dependency unit for treatment of supposed amphetamine dependence | He regularly obtained a white powder believed to be a high-purity amphetamine from a contact who apparently worked in a laboratory |
| Forrester, 2011 | Retrospective study | N= 3,421 (M: 60.4%) | Mean age= 13–19 yrs, (69.0%) | NR | NR | Coricidin® (53.6%);  Vicks® and other  Proctor & Gamble products  (13.0%); Robitussin® (11.4%); and  Delsym® (5.2%), oral | Tachycardia (31,9%); drowsiness/lethargy (25,2%); hypertension (14,9%); mydriasis (8,4%); vomiting (7,5%); dizziness (5,7%); ataxia (5,4%); nausea (3,7%) | Agitation/irritability (10,1%); confusion (8,5%); psychotic symptoms (5,7%) | DXM+ chlorpheniramine (52.2%); DXM alone  (11.5%); DXM+ acetaminophen+ doxylamine+ pseudoephedrine (10.2%);  DXM+ guaifenesin (9.4%) | A potentially serious outcome was assigned in 46.2% of the cases and 83.1% were managed at a health care facility; death (0,1%) | Intentional misuse or abuse ingestions of DXM-containing products reported to Texas Poison Centers (2000–2009) |
| Ganetsky et al., 2007 | Case report | N=1 (M) | Age= 18 yrs | NR | NR | 960mg of DXM, oral | Serotonin syndrome, including the following symptoms: autonomic instability, neuromuscular hyperactivity, mydriasis, tachycardia, diaphoresis, ocular clonus, and hyper-reflexia | Psychomotor agitation | Chlorpheniramine | In the ED he was sedated with IV lorazepam followed by a continuous propofol infusion which rapidly normalized agitation, neuromuscular hyperactivity, and autonomic instability; discharged in stable condition |  |
| Ghosh, 2011 | Case series | N=3 (2M, 1 F) | Mean age = 21,3 yrs | Case 1: NR; Case 2: tobacco smoker (40 cigarettes daily); Case 3: tobacco smoker (6 cigarettes per day), occasional past use of sleeping pills and hard drugs | Case 1, 2: NR | Case 1: 600 mg of DXM daily, oral; Case 2: cough mixtures use started 2 yrs before for sinusitis and cough, and then increased the amount to 1bottle daily (890mg of DXM); Case 3: DXM, codeine and DPH containing cough mixtures (up to 2bottles = 1,780mg of DXM)/ month | Case 1, 2, 3: NR | Case 1: paranoid delusions and visual and auditory hallucinations; Case 2: psychotic symptoms including delusions and auditory hallucinations, drowsiness, confusion. Case 2: she denied any psychotic or mood symptoms, but she appeared elated and unaware of her addiction problem; Case 3: long-term use of cough mixtures containing DXM, codeine and DPH  . | None | Case 1: treated for two days with light sedation (hydroxyzine) and subsequently discharged with advice to see the Addiction Medicine Department; Case 2: patient treated with light sedation (hydroxyzine) and discharged with outpatient appointments to see the Addiction Counsellor and Psychiatrist; Case 3: patient's condition stabilized, and he was discharged agreeing to be followed up by the Addiction Counsellor and Psychiatrist | Case 2 was implicated in doctor shopping behaviours and was fined for stealing cough mixture from the clinic where she worked |
| Hapangama and Kuruppuarachchi, 2011 | Case series | N=5 (M) | Mean age= 20.4 yrs | NR | NR | >120mg DXM daily, oral | NR | DXM abuse associated with the following symptoms: elevated mood; irritability; auditory and visual hallucinations, paranoid, and grandiose delusions; social withdrawal; dissociation and depersonalization | NR | Treated with antipsychotics and behaviour therapy programs |  |
| Helfer and Kim, 2001 | Case report | N=1 (M) | Age= 22 yrs | History of SUD (polysubstance) | None | 20-22oz of RubitussinDM® (590-826mg of DXM), oral | Polyuria; vomiting, abdominal gas, difficulty in focusing, slurred speech, ataxia | Severe dysphoria, sleeping disorder | None | Arrived at an army hospital emergency room complaining of severe dysphoria due to failing an army training examination and acute marital discord |  |
| Hendrickson and Cloutier, 2007 | Case report | N=1 (M) | Age= 20 yrs | NR | NR | 1g of ‘Crystal dex’, oral | Unresponsiveness, tachycardia (99bpm), hypotension (70/30mmHg), mydriasis, dried skin, flushing; approximately 5 hours after admission, he showed psychomotor agitation, tachycardia (120bpm), hypertension (202/88mmHg) | NR | None | Brought to the ED, he was treated with naloxone and flumazenil IV, and intubated; midazolam for agitation | DXM extracted through an acid-base extraction technique in order to produce a purified free-base ‘Crystal dex’ separated from unwanted  guaifenesin, colouring agents, sweeteners, and alcohol that are typically included in combination cold preparations |
| Hinsberger et al., 1994 | Case report | N=1 (M) | Age= 39 yrs | SUD (alcohol) | NR | Oral | Cognitive deterioration (SPECT); excessive central alpha activity (EEG); ataxia, bilateral nystagmus, dysnomia, severe muscle weakness and trance-like spells | Mood fluctuations; aggressive and disruptive behaviour; poor attention span; intense suicidal ideation; restlessness; insomnia; delusions; and visual hallucination | Alcohol | Hospitalization in acute maniac state | Progressive cognitive deterioration and worsening of psychiatric symptoms due to the long-term drug abuse |
| Iaboni and Aronowitz, 1995 | Case report | N=1 (M) | Age= 37 yrs | Chronic paranoid schizophrenia; SUD (alcohol) | None | DXM-containing syrup (240-720mg of DXM), oral | Diarrhea, nausea, insomnia, vertigo, vomiting, diaphoresis, and erectile dysfunction | Pleasure; heightened awareness to environmental stimuli; altered time perception; thought broadcasting; panic; agoraphobia; craving after long-term use | Alcohol | Admitted to alcohol rehabilitation unit |  |
| Jamison and Vasudeva, 2009 | Case report | N=1 (F) | Age= 60 yrs | NR | Hypertension | 120mg of DXM per day, oral | NR | Substance-induced delirium with psychomotor agitation, religious delusions, visual and olfactory hallucinations, aggressiveness | Propoxyphene, hydrocodone | Treated with lorazepam, olanzapine, and aripiprazole; discharged in stable condition | On treatment with atenolol, furosemide, and esomeprazole |
| Kaplan et al., 2011 | Case report | N=1 (M) | Age= 27 yrs | SUD (alcohol) | NR | Four bottles of Robitussin ® (1,920 mg of DXM) and ethanol, oral | Tachycardia (HR: 101bpm), QTC interval prolonged (514 ms) | NR | Alcohol | Found altered in a bathroom, he was conducted to the ED. Discharged after resolution of symptomatology |  |
| Kimber and Thompson, 2015 | Case report | N=1 (M) | Age= 42 yrs | SUD (alcohol and tobacco), depression | History of recurrent injuries secondary to falling while intoxicated | Habitual use of DXM cough syrup for 7 yrs (daily intake 600mg of DXM), oral | Movement disorders associated to DXM: delayed-onset segmental dystonia, involuntary facial movements, including frowning, blepharospasm, trismus, and grimacing | Chronic use of DXM to treat depression, as well as to suppress cravings for alcohol and tobacco | None | Started botulinum toxin treatment, with significant improvement in the severity of the dystonia over 12 months |  |
| Kirages et al., 2003 | Case series | N=2 (F) | Mean age: 16 yrs | Case 1: depression and bipolar disorder; case 2: depression and SUD (cannabis) | Cases 1-2: none | Case 1: 20 tablets of Coricidin® (200mg of DXM), oral; case 2: 50 tablets of Coricidin® (500mg of DXM), oral | Case 1: HR 150bpm, BP 170/100mmHg, and RR 18 breaths/min; mydriasis, horizontal nystagmus, hypoactive bowel sounds; case 2: nausea, vomiting, abdominal discomfort, urinary retention | Case 1: somnolence, confusion, and anxiety; case 2: NR | Case 1: fluoxetine and DPH; case 2: acetaminophen and chlorpheniramine | Case 1: found sleeping in front of her home and conducted to the ED where she received activated charcoal, IV fluids, and discharged asymptomatic after 72hours with outpatient follow-up; case 2: presented to the ED and treated with oral N-acetylcysteine, then transferred to a pediatric care center where lactulose and fresh-frozen plasma were administered. She was discharged after 7-day hospital stay | Case 1: on treatment with olanzapine and fluoxetine |
| Linn et al., 2014 | Case report | N=1 (M) | Age= 30 yrs | SUD (tobacco Oxycodone and alcohol), depression | Insulin dependent Diabetes Mellitus, hypertension, persistent nasal congestion after nasal fracture | Daily use of 1440 to 1800mg of DXM for six months, oral | NR | Feeling “dumb and numb” like “did not have to think [about] problems”; he described marked fatigue and depressed mood on days that he did not ingest DXM, but he denied physical signs and symptoms of drug withdrawal | None | He underwent a scheduled elective sept rhinoplasty. Postoperative pain was treated using intravenous hydromorphone (1.2 mg) in the post anesthesia care unit and was subsequently managed using oral hydrocodone with acetaminophen after the patient was discharged from the hospital later that day |  |
| Logan, 2009 | Case series | N=12 (10 M, 2 F) | Mean age 26,4 yrs | NR | NR | 480-1,440mg of DXM (Coricidin®), oral | Ataxia, slow or slurred speech, tachycardia and hypertension, tremors | CNS depressant intoxication, with impairment in the driving and involvement in collisions | Cannabis, guaifenesin, alcohol | NR | A series of cases regarding 12 drivers arrested for driving under the influence of the effects of DXM-containing products |
| Logan et al., 2012 | Case series | N=5 (5 M) | Mean age: 21yrs | Case 1-2: NR; Case 3: SUD (DXM+ chlorpheniramine, LSD, and cannabis); Case 4: NR; Case 5: history of SUD (opioids) | Case 1,2, 3,4, 5: NR | Case 1: intensive DXM use over a period of 12 months (doses of 600–900mg daily); Case 2: ingested 16 tablets of Coricidin® (430mg of DXM); Case 3: 22 Coricidin® pills (660mg); Case 4: 164 pills of Corcidin® (4,920mg of DXM); Case 5: 300–500mg of DXM daily | Case 1, 2, 3, 4: NR | Case 1: DXM-induced psychotic symptoms (paranoia and auditory hallucinations) and altered behaviour with auto/hetero aggressiveness; Case 2: he attempted suicide due to psychotic symptoms, attempting to exit the window of the fifth-floor room. He was restrained by his friends, but soon after one of them noticed he was not breathing and called paramedics, who pronounced him dead; Case 3: found dead at home (hanging by the neck from the closet doorknob); Case 4: found dead at home; Case 5: DXM-induced psychosis with abnormal behaviour and aggressiveness | Case 1: none; Case 2: chlorpheniramine, cannabis; Case 3: cannabis; Case 4: chlorpheniramine; Case 5: NR | Case 1: He wounded himself to death; Case 2: drug-related death; Case 3, 4: suicide; Case 5: after 2 days of symptomatic treatment, his mental state cleared and his behaviour returned to baseline |  |
| Majlesi et al., 2011 | Case report | N=1 (F) | Age= 19 yrs | Bipolar disorder; SUD (alcohol and DXM) | NR | Unspecified dosage of DXM, oral | Seizure secondary to DXM abuse; tachycardia, horizontal nystagmus | Confusion, amnesia | Drug screen negative | Brought to the ED due to ‘‘shaking’’ noted by witnesses. She was observed for 24 hours and discharged without sequelae, referred for outpatient detoxification and substance abuse treatment | Prescribed lamotrigine and aripiprazole |
| Marsh et al., 1997 | Case report | N=1 (F) | Age= 14 yrs | Bulimia; depression; SUD (alcohol, cannabis, cocaine, and LSD) | None | >237mg of DXM, oral | NR | Poor concentration, blackouts, and visual hallucinations | Alcohol, cannabis | She was hospitalized for 30days for intensive psychiatric treatment and started on Prozac 20 mg, and individual/group-therapy for the treatment of her eating disorder and substance abuse | Long-Term abuse of DXM to lose weight |
| Martinak et al., 2017 | Case report | N=1 (F) | Age= 40 yrs | SUD (alcohol, cannabis, LSD, opioid, benzodiazepine, amphetamines); previous psychotic episode, PTSD, insomnia, depression | NR | Coricidin™  Cough and Cold (1,080-4,000mg/day of DXM total) | NR | DXM use disorder with DXM induced psychotic disorder: irritability; mood lability; suspiciousness; paranoid delusion; aggressivity | Alcohol | Symptoms remitted following treatment with an antipsychotic and mood stabilizer (olanzapine and valproate) |  |
| Medows et al., 2020 | Case Report | N=1 (F) | Age= 15 yrs | Major depressive disorder and anxiety disorder | None | Misuse of Coricidin® ( acetaminophen, chlorpheniramine, and DXM), oral | Abdominal pain, emesis, slightly elevated BP and RR | None | None | Brough to the ED with a primary concern of abdominal pain. Discharged on hospital day 6 with drug-induced liver failure and concomitant pancreatitis |  |
| Miller, 2005 | Case report | N=1 (M) | Age= 18 yrs | Dysthymia | None | Started from 240mg daily of DXM syrup, then preferring DXM-containing Coricidin® pills (easier to carry on, to steal from stores and to titrate), max dose 480 mg daily, oral | NR | ‘‘Floaty’’ feeling, sense of euphoria, mild dissociation, and altered perception of time; dysphoria, restlessness and craving after abruptly stopping use | None | Naltrexone was used as an antirelapse agent for DXM dependence | DXM abuse began at  the age of 17, upon contacts from friends and the Internet |
| Modi et al., 2013 | Case report | N=1 (F) | Age= 46 yrs | Mood disorder and SUD (methamphetamine, oxycodone) | NR | Four bottles of cough syrup containing DXM (10 mg each) | At the time of admission, her BP was 134/82 mmHg, HR 133bpm, and RR 16 breaths/min | Substance-induced psychosis with paranoia, auditory hallucination, abnormal behaviour with auto/hetero aggressiveness, insomnia | Guaifenesin; patient’s urine drug screen, and alcohol level were negative | In the emergency care setting, the patient required surgical intervention for multiple deep lacerations on her left upper arm and fingers. She was then medically cleared and transferred to the Psychiatric Crisis Unit, where she was observed for 24 hours and then discharged calm and cooperative without hallucinations or delusional beliefs, to police for alleged attempted homicide | The patient stated that she was trying to self-medicate at home to treat the withdrawal symptoms from oxycodone |
| Monks et al., 2020 | Case report | N=1 (F) | Age= 47 yrs | History of SUD and depression | NR | 50 pills of Robitussin® (20mg DXM per pill), oral | Tachycardia (112 bpm) and hypertension (149/85 mmHg); hyperchloremia (125 mmol/L, normal 98–107), decreased bicarbonate (13 mmol/L, normal 22–30), bromide markedly elevated (130 mg/L, normal 1.4–8.8 mg/L), anion gap of 1; venous blood gas revealed acidosis with pH of 7.19 | Emotional lability | None | Presented to the ED for a drug overdose; required fluids and sodium bicarbonate to solve the acidosis. Admitted to an inpatient psychiatric unit for 4 days, where she was started on bupropion for depression |  |
| Murray and Brewerton, 1993 | Case series | N=2 (M) | Mean age 15±1.4 yrs | Case 1: Depression; SUD (cannabis and alcohol); case 2: NR | Case 1: None; case 2: seizures | Case 1: 3 bottles of Robitussin® (708mg of DXM), oral; case 2: 1 bottle of Robitussin® (118mg of DXM), oral | Case 1: NR; case 2: seizure-like episode, hypertension, tachycardia | Case 1: due to self-destructive behaviour and self-mutilation; aggressive behavior, disoriented; case 2: unresponsiveness, incoherence | None | Case 1: admitted to the psychiatric hospital and discharged after symptoms resolution; case 2: brought to the ED where he was treated with naloxone IV, gastric lavage, charcoal with magnesium citrate and after discharged sent to the mental health center |  |
| Mutschler et al., 2010 | Case report | N=1 (M) | Age= 44 yrs | SUD (alcohol) | NR | 1,800mg of DXM daily, oral | NR | Dependence on DXM through 6 yrs of abuse; after the abrupt interruption he developed a vegetative withdrawal syndrome consisting of craving, diaphoresis, nausea, hypertension, and tachycardia | None | The patient attended the outpatient addiction clinic where he completed the behavioral therapy–based detoxification program (21 treatment days) and continued the treatment as an outpatient |  |
| Navarro et al., 2006 | Case report | N=1 (M) | Age= 22 yrs | Bipolar disorder and alcohol abuse | None | Several bottles of DXM-containing  cough syrup, oral | Serotonin syndrome with hyperthermia, tremor, hypertension, tachycardia, palpitations, , diaphoresis, nystagmus, hyperreflexia, clonus, restlessness, akathisia | Psychomotor agitation | None | Brought to the ED, he was treated with oral propranolol and lorazepam to manage BP and control complaints of restlessness and akathisia. Symptoms resolved after several days; discharged in stable condition | On treatment with lithium, fluoxetine, clonazepam |
| Nordt, 1998 | Case series | N=2 (M) | Mean age=17,5yrs (SD=0,5) | None | None | Oral | Diaphoresis, incoordination, mydriasis, drowsiness, nystagmus, tachycardia, hypertension, tachypnoea | Agitation; alert state | None | Case 1: in the ED treated with lorazepam IV for agitation, and then discharged the following day without symptoms; Case 2: a single dose of activated charcoal was given and after observation in the ED he was discharged |  |
| Oakland et al., 2016 | Case report | N=1 (M) | Age= 29 yrs | Bipolar I disorder, anxiety, and a prior suicide attempt | NR | 32 capsules of Coricidin Cough and  Cold®, containing DXM (960mg) and chlorpheniramine, oral | Serotonin syndrome, including disorientation, agitation, diaphoresis, confusion, tachycardia, hypertension, clonus, and rigidity | Psychomotor agitation, confusion | Chlorpheniramine | Discontinued buspirone and duloxetine due to their serotonergic mechanism; he was administered lorazepam as needed and IV fluids. Transferred to psychiatry unit, improved with no new medications, and discharged within two days | On treatment with buspirone, duloxetine and lurasidone |
| Orrel, 1986 | Case report | N=1 (M) | Age= 37 yrs | SUD, schizophrenia | None | 2 bottles of DXM-containing syrup several times a week, oral | None | Anxiety, irritability, aggressiveness | None | Admitted to the psychiatric department and discharged after; two weeks of antipsychotic medication |  |
| Osterhoudt and Miloradovich, 2010 | Case report | N=1 (M) | Age= 15 yrs | NR | NR | He consumed 3 pills containing DXM, oral | Nausea, vomiting, blurred vision, tachycardia (HR: 124bpm), mydriasis, horizontal nystagmus, mild tremor of the hands | Alertness, slurred speech, confusion | None | Admitted to the ED due to vomiting and diminished alertness. The boy’s condition improved during the subsequent 12 hours after intravenous hydration and ondansetron, and he recovered completely | The pills were named ‘snurf’, purchased from the Internet, and marketed as a legal high |
| Polles and Griffith, 1996 | Case report | N=1 (M) | Age= 43 yrs | Depression, SUD (opioid) in remission | NR | Unspecified dose of Delsym Cough Formula®, oral | None | Paranoid delirium with bizarre behaviour; manic state with euphoria, insomnia, endless energy, excessive expenses, tachypsychism, and auditory hallucinations | None | Brought to the hospital by police after he aroused his neighbour by shouting for help. They discovered him half-clothed wielding a gun in a frantic effort lo free his wife whom he believed was held hostage by intruders. Admitted to the ED treated with IM haloperidol. Manic symptoms and psychotic thought steadily diminished after DXM discontinuation |  |
| Ritter et al., 2019 | Retrospective, cohort analysis | N=203 patients were diagnosed with DXM toxicity (F: 112, 55.2%) | Mean age 28.0±20.7 yrs (age range 1-97) (SD=NA) | N=95 (46.8%) SUD | NR | Oral | Tachycardia (47.0%), somnolence (38.9%), dizziness (23.2%), confusion (22.1%), trembling (19.2%), gastrointestinal effects (18.2%), blurry vision (14.3%), palpitations (10.3%), dry mouth (8.4%), ataxia (4.9%), diaphoresis (3.9%), fever (3.0%), dystonia (1.0%), urinary retention (1.0 %) | Anxiety (18.2%), hallucinations (11,8%), loss of consciousness (9.4%), delusions | Alcohol, opiates, and cannabis | Poor outcomes were related to both DXM and the presence of co-ingestants commonly found in OTC cough and cold products, and included anticholinergic toxicity, respiratory depression, serotonin syndrome, toxic encephalopathy, and acetaminophen toxicity. Prognosis depended on patient age, comorbidity, and polysubstance abuse. There were no fatalities | Patients seen at 7 EDs in Michigan over a 15-year study period (January 2004-December 2018) |
| Roberge et al., 1999 | Case report | N=1 (M) | Age= 22yrs | NR | NR | 33.75mg of DXM, oral | NR | Hyper irritability, drug-induced agitated psychosis, and ataxia | Pseudoephedrine | Discharged uneventfully after supportive care |  |
| Roy et al., 2013 | Case report | N=1 (F) | Age= 45 yrs | Opioid dependence, major depressive disorder, and obsessive-compulsive disorder |  | 800mg per day, oral | NR | Euphoria and stimulating effect after ingestion; multiple psychotic episodes while taking high doses of DXM resulting in at least 4 emergency department visits; long-term use was associated with intense craving, tolerance, and withdrawal symptoms, including severe fatigue, depression, and restlessness | None | Treatment with topiramate resulted in complete resolution of her craving | Common pathways of addiction to DXM and alcohol through the NMDA system have been suggested |
| Sharma et al., 2005 | Case report | N=1 (M) | Age= 54 yrs | None | Myocardial infarction and lymphoma | 240mg, oral | NR | Acute psychosis with paranoid thoughts and auditory hallucinations | Chlorpheniramine | Admitted on the ED and then the inpatient psychiatric facility where he was treated with haloperidol 5 mg IM; symptoms resolved over 2 days | On treatment with aspirin, lisinopril,  and clopidogrel |
| Stanciu and Penders, 2015 | Case report | N=1 (M) | Age= 20yrs | NR | NR | 600mg, oral | NR | Substance-induced manic toxidrome with  psychotic features (including auditory hallucinations, delusions, agitation, pressured speech, and grandiosity), with onset during intoxication | Chlorpheniramine | Risperidone and lorazepam administered; discharged in stable condition and symptom-free at four-month follow-up |  |
| Tsang and Au, 2012 | Case series | N=57 cough mixture abusers  (M/F=46:11) | Median age: 31 yrs; age range:  19-49 yrs | Cases 1-3: SUD, (alcohol, benzodiazepines, ketamine, and cough mixtures) cases 1-2: schizophrenia; case 3: delusional disorder | Rhabdomyolysis | Case 1: codeine-containing cough mixtures, oral; case 2: abuse of cough mixtures containing ephedrine, DXM and  codeine (2 bottles/daily), oral; case 3: NR (tox screening revealed ephedrine and DXM) | Case 1: Uncoordinated limb movements myalgia, and elevated creatinine phosphokinase level (58 500 IU/L; reference level, <250 IU/L); case 2: rigidity, increased creatinine phosphokinase, and fever; case 3: myalgia, proximal muscle weakness and swelling and elevated creatinine phosphokinase level (3,200UI/l) with myoglobinuria | Cases 1-2: confusion; case 3: NR | Case 1: Midazolam, Ketamine, DXM | Case 1: hydrated, and discharged after a week, without neurological sequelae; case 2: antipsychotics were withheld, and folate supplements used; case 3: he improved after hydration and vitamin supplements, but was lost to hospital and out-reach clinic follow-up | On a sample of 57 cough mixture abusers, the report focused on three patients alone, found to have a history of rhabdomyolysis and to be on treatment with antipsychotics |
| Walker and Yatham, 1993 | Case report | N=1 (M) | Age= 40 yrs | None | None | Benylin DM 400ml daily (600mg of DXM), oral | None | Mania | None | He was treated with small doses of haloperidol with rapid resolution of  symptoms. While in hospital, he experienced craving for DXM |  |
| Wolfe and Caravati, 1995 | Case report | N=1 (M) | Age= 23 yrs | SUD (alcohol) | None | 12-ounce bottles of cough syrup (Robitussin DM®), 2,160 mg, oral | Flushing; diaphoresis; horizontal and vertical nystagmus; muscles fasciculations and hypertonicity; marked ataxia | Psychomotor agitation; hallucinations; confused speech | Alcohol | Transported via ambulance to the hospital after being found in a snowbank "agitated and hallucinating." Treated with 50g activated charcoal orally and IV thiamine and naloxone | Attending group therapy for DXM-addiction |
| Ying-wei et al., 2017 | Prospective study | N= 56 (M)  (38 DXM dependent subjects vs 18 HC) | Mean age: DXM dependent subjects 23.7 yrs ys HC 24.0 yrs | NR | NR | Oral | NR | At the MRI DXM dependents exhibited compared with HC significantly increased cortical thickness in the PreC, DLPFC.L, IPL.L, PreCG.R, LOC.R, ITC.R, lOFC.R and TTG.R; and increased subcortical volumes of the right thalamus and right pallidum. There was a significant correlation between initial age of DXM use and cortical thickness of the DLPFC.L and PreCG.R. A significant correlation was also found between cortical thickness of the DLPFC.L and impulsive behaviour | None | NR |  |
| Ziaee et al., 2005 | Case series | N=53 (M:.48, F: 5) volunteers who had consumed DXM | Mean age 23.4 yrs, (SD = 1.83). | Depression, anxiety, occasional drug abusers | NR | 75-2,700mg, oral | Autonomic side effects, e.g., sweating, tachycardia, fatigue, tachypnea, flushing; gastrointestinal effects, e.g., nausea, vomiting, diarrhea, constipation; neurological effects, e.g., dyskinesia, speech disorder, dizziness, mydriasis, photophobia, blurred vision, diplopia, imbalance, dysaphia, tremor; other side effects, e.g., itching, fatigue, urticaria, myalgia, altered libido | Euphoria, trance, apathy, laughing, tongue biting, auditory and visual hallucinations, insomnia, nightmares, anhedonia, dysmnesia, hypervigilance, attention deficit, learning impairment, flashback, panic disorder, hyperactivity | Alcohol (96.2%), cannabis (88.7%), sedatives (71.7%), LSD (67.9%),morphine 54.7%, ecstasy (52.8%), cocaine (30.2%), heroin (24.5%), phencyclidine (20.7%), ketamine (20.7%), others (24.5%) | No medical support needed | Causes of repeated DXM abuse were  psychological dependency (46.5%), and recreational abuse (32.6%). |
| Dimenhydrinate (DH) | | | | | | | | | | | |
| Brown and Sigmundson, 1969 | Case report | N=1 (M) | Age= 18 yrs | None | None | Unspecified dosage, oral | Mydriasis, tachycardia, hypertension, urinary incontinence, difficult speech, flushing, restlessness, dystonia | Drug-induced delirium with paranoia, thought incoherence, emotional lability, agitation, visual and auditory hallucinations, anxiety, | None | ED treated with IV chlordiazepoxide and benztropine |  |
| Kaya, 2014 | Case report | N=1 (F) | Age= 33 yrs | Depression | None | 300mg daily, oral | Withdrawal symptoms included: nausea, vomiting, hand tremors, perspiration, drowsiness, headache, poor appetite | Withdrawal symptoms included irritability and anxiety | None | Referred to the outpatient psychiatry clinic by an emergency physician with withdrawal symptoms after stopping DH for three days. DH was started again with a decreased dose (250 mg/day) because of her severe withdrawal symptoms. Then, DH dose was gradually tapered down and stopped within five weeks | Firstly advised to use DH 50 mg/day for emesis early in her second pregnancy by her primary care physician, then she continued to use the drug for 12 yrs at a higher dose without a prescription |
| Malcolm and Miller, 1972 | Case series | N=2 (M) | Mean age 21 yrs (SD=1) | Case 1: SUD (LSD, cannabis, mescaline, barbiturates, and cocaine); Case2: SUD (cannabis, mescaline, LSD) | Cases 1-2: none | Cases 1-2: 16 tablets (800mg), oral | Case 1: dried mouth and sleepiness; case 2: lethargy | Case 1: anxiety; visual and auditory hallucinations; case 2: paranoia, visual hallucinations | Cases 1-2: none | Cases 1-2: NR |  |
| Oliver and Stenn, 1993 | Case report | N=1 (F) | Age= 39 yrs | Dependent personality disorder and SUD (opiates and benzodiazepines) | Multiple gastrointestinal operations, multiple sclerosis, and eosinophilic myositis | IM | None | She was requesting DH to control her nausea well beyond the period of gastric adjustment following surgery. Furthermore, she would often "stack" her medications by simultaneously requesting her opiates, benzodiazepines, and DH. Finally, she was resistant to either 1) tapering the dosage or frequency of the IM DH, or 2) conversion to equivalent dosages in either the oral or rectal form | Opiates and benzodiazepines | Referred to psychiatry consultation-liaison service for management of chronic pain problem; discharged from the hospital with outpatient follow-up |  |
| Prost and Millson, 2004 | Case series | N=2 (M:1, F:1) | Case 1: 35yrs; Case 2: NR | Case 1: Schizophrenia; Case 2: Schizophrenia, SUD (cocaine) | Cases1: seizures; Case2: none | Case 1: 5,000mg of DH, oral; Case 2: 3,000mg of DH, oral | Case 1: generalized seizures; Case 2: NR | Case 1: euphoria, getting ‘high’; Case 2: stimulant effects | None |  | Clozapine helped to reduce craving for DH |
| Rowe et al., 1997 | Case series | N=8 (F) | age= 14-17yrs | Cases 1-8: none | Cases 1-8: none | Cases 1-8: 15tablets (750mg), oral | Case 1: ataxia, mild tachycardia; cases 2-3: asymptomatic; cases 4-8: unsteady gaits, ataxia, tachycardia | Case 1: visual hallucinations, disorientation, confusion, slurred speech and abnormal behaviour; cases 2-3: asymptomatic; cases 4-8: NR | Cases 1-3: cannabis; cases 4-8: none | Case 1: admitted to the ED, no treatment required; cases 2-3: no medical support was needed; cases 4-8 brought to the ED by police officers; symptom resolution was rapid without treatment |  |
| Wen et al., 2019 | Case report | N=1 F) | Age= Late forties | Bipolar disorder, SUD | Familiar  Mediterranean fever and fibromyalgia | 100 -200mg of DH hourly for its euphoric and energetic effects (upwards of 2400mg/day) | Withdrawal symptoms included nausea, vomiting, sedation, headaches, dizziness, anxiety, agitation, and muscle stiffness | Psychomotor slowing with difficult speech and delayed verbal responses. Her insight and judgment were unable to be assessed due to her sedation | None | Admitted to the hospital for withdrawal management (benztropine and lorazepam) The patient was discharged to a community inpatient rehabilitation center | She was prescribed 60 mg of lurasidone for mood stabilization, and 24 mg of buprenorphine/naloxone for her chronic pain |
| White et al., 2015 | Case report | N=1 (F) | Age= 38 yrs | Chronic schizophrenia, SUD (cocaine, methamphetamine, and cannabis) | Gastroesophageal reflux disease | Daily use of 20 or 30 tablets of DH (1,000-1,500mg), oral | Tachycardia; dyskinesia; gastrointestinal symptoms | Persecutory delusion; auditory, visual, and tactile hallucinations; irritability; disorientation and disorganized behaviour; | None | Referred to the B. C. Psychosis Program for treatment-resistant psychosis. Discharged and 3 months later, stable on the same medications | On treatment with haloperidol 1 mg daily, zuclopenthixol dec 250 mg IM every 2 weeks, propranolol 20 mg twice daily, and citalopram 40 mg daily |
| Diphenhydramine (DPH) | | | | | | | | | | | |
| Bonham and Birkmayer, 2009 | Case Report | N=1 (F) | Age= 34 yrs | Chronic undifferentiated schizophrenia and SUD (cocaine) | NR | Oral | Withdrawal symptoms consisted in bowel and bladder incontinence, hyperpyrexia, hypertension, hypertonia in her upper extremities, and extrapyramidal symptoms | Concretism, ideas of reference, thought insertion, and chronic auditory hallucinations. Her insight and judgment were impaired | None | She remained as an inpatient on the psychiatric ward for 28 days. The DPH was stopped; patient was treated with benztropine, fluphenazine, and quetiapine |  |
| Chen et al., 2014 | Case report | N=1 (F) | Age= 49 yrs | Recurrent major depressive episodes, SUD (alcohol) | NR | Initially prescribed 30mg daily, then dosage increased to 450mg daily, IM | ECG revealed sinus rhythm and prolonged QTc =503ms | After use: relaxation, calmness, sleep improvement; due to long-term administration, craving and withdrawal symptoms including anxiety, irritability, poor attention, and rebounding insomnia | None | Clonazepam, flurazepam, zolpidem | Doctor shopping |
| Cox et al., 2001 | Case report | N=1 (M) | Age= 34 yrs | Dysthymia, panic attacks, occasional auditory pseudo hallucinations, SUD (alcohol and cannabis) | None | 4–6 bottles of Benylin for  Chesty Cough ® (3,360-5,040mg of DPH) weekly for 5 months, oral | Mydriasis and symptomatic postural hypotension | NR | Alcohol | NR | On treatment with diazepam, chlorpromazine, and lofepramine |
| Dinndorf et al., 1998 | Case series | N=5 (M:2, F: 3) | Mean age 13.4yrs (SD=4.6) | Cases 1-5: none | Case 1: leukemia; case 2:  Ewing’s sarcoma; case 3: dyskeratosis congenita, aplastic anemia, gastritis, esophagitis, and chronic pain; case 4: myelogenous  leukemia and nasopharyngeal and CNS aspergillosis; case 5: sarcoma | Case 1: unspecified dose, oral; cases 2-4: unspecified dose, IV; case 5: 50 mg of DPH/ 2 hours while awake | Case 1: NR; case 2: choreoathetoid movements and urinary retention; case 3: choreoathetoid movements and dyskinesia; case 4: NR; case 5: difficulty urinating, excessive dry mouth, and tremors | Case 1: She reported that she habitually used DPH seeking the sedative effect of the drug; case 2: psychomotor agitation; case 3: restlessness and disorientation; case 4: craving; case 5: NR | Case 1: none; cases 2-4: none; case 5: lorazepam | Case 1: NR; case 2: DPH gradually discontinued and replaced with dronabinol for antiemetic relief; case 3: DPH discontinued, but continued drug-seeking behavior for this medication over time; case 4: despite parental control on drugs, he was hiding DPD and continued to request that caregivers administer DPH by rapid bolus; case 5: DPH and lorazepam gradually discontinued; referred for formal psychologic therapy | Case 2: on treatment with IV morphine for pain and IV DPH for nausea; case 3: on treatment with opioids for pain and DPH for itching; case 4: on treatment with amphotericin, IV DPH and meperidine; case 5: receiving IV patient-controlled morphine for pain and IV lorazepam and DPH as antiemetics |
| Feldman and Behar, 1986 | Case report | N=1 (M) | Age=34 yrs | Chronic residual schizophrenia | SUD (alcohol) | 1,600mg daily, oral | Dry mouth, urinary hesitancy, tachycardia | Slight euphoria and mild grandiosity without psychotic features | Thiothixene | Tapering of DPH was instituted, appearing craving, rebound insomnia and restlessness; patient was discharged on day 15 without symptoms | Initiated use of DPH six months prior to admission to assist with initial insomnia |
| Gracious et al., 2010 | Case report | N=1 (F) | Age=15 yrs | Bipolar disorder and anxiety disorder | SUD (alcohol, opioids, and cannabis) | >400mg of DPH daily, oral | Withdrawal symptoms included: tachycardia, tachypnea, hypertension, nausea, nasal congestion | During discontinuation, she showed craving, irritability, and drug-seeking behaviours | NR | During the hospitalization DPH was gradually discontinued, and withdrawal symptoms managed with naltrexone | Her mother reported frequently empty boxes of DPH were found in the patient's room |
| Hermann and Bassetti, 2005 | Case report | N=1 (F) | Age= 36 yrs | None | History of migraine | 2,000mg of DPH, oral | Reversible opsoclonus with irregular conjugated nystagmus; mydriasis; ataxia; flushing | Agitation; confusion; anxiety; irritability | NR | In the ED treated with clonazepam (3 mg/day) and then discharged after symptoms resolution | Pharmacy confirmed repeated purchases of DPH during the last few weeks |
| Phillips et al., 2014 | Case report | N=1 (F) | Age= 13 yrs | Previous Suicides | NR | Oral | Antimuscarinic toxicity with mydriasis, flushing, hyperthermia, myoclonus, and rigidity, urinary retention, hypertension (143/54 mmHg), tachycardia (HR 160bpm), and tachypnea (RR 46 breaths/min) | Antimuscarinic toxicity with hallucinations and severe psychomotor agitation | Bupropion, citalopram, acetaminophen, omeprazole, and naproxen | Brought to the ED following a polydrug overdose; several doses of lorazepam and physostigmine produced resolution of hallucinations and attenuation of the antimuscarinic symptoms. GABAergic agents were used later in the hospital course for presumed symptoms of serotonergic and adrenergic toxicity after resolution of antimuscarinic effects |  |
| Saran et al., 2016 | Case Report | N=1 (M) | Age= 21 yrs | Previous drug-related admissions due to intoxications | None | Oral | Withdrawal syndrome from abrupt cessation of DPH, including tremors, seizures, hypersalivation, diaphoresis, flushing, hypomimia, hypophonia, dysarthria, mydriasis | Psychosis | None | Treated with levetiracetam and phenytoin along with haloperidol and benzodiazepines for symptomatic management of ongoing psychosis. The IV administration of DPH resulted in immediate improvement. He was then started on oral DPH, finally gradually reduced |  |
| Smith and Davis, 1984 | Case report | N=5 (M) | Age range 14 to 19 yrs; | NR | NR | Dose was gradually increased, from 150-225mg up to 450-750mg, IV | NR | After use: sedation, drowsiness, confusion, clouded sensorium, uncommunicativeness, and stupor; a minor abstinence syndrome included agitation, dysphoria, sleep disturbances, difficulty in concentrating, emotional lability, and irritability | Butorphanol | Admitted to the clinical service of a community mental health center; 1 death recorded due to respiratory failure after an unusually high drug intake |  |
| Sundararaghavan and Suarez, 2004 | Case report | N=1 (F) | Age= 17 yrs | None | Osteogenic sarcoma and history of deep vein thrombosis | Self-injection of 4x50mg crushed DPH capsules  into her central venous catheter | Signs of shock: cool and clammy extremities; labored breathing; incoherent speech; tachycardia (180-220bpm); hypotension (70/30mmHg); perioral cyanosis; weak peripheral pulses; metabolic acidosis | NR | None | Found on the floor, hospitalized, and treated symptomatically with oxygen and diuretics over a period of weeks. Fluid resuscitation and norepinephrine infusion; anticoagulation with heparin was also initiated to prevent further pulmonary arterial thrombosis. Short-term and long-term therapy included child psychologic services | The subject attempted  suicide |
| Tennant, 1973 | Retrospective review (Jan-Jun 1972) | N=67 (M) | Age range: 18 to 24 yrs | NR | None | Unspecified dose, oral | Overdose cases with stuporous or comatose condition, tremor of hands and legs, and muscular rigidity; physical dependence, with withdrawal signs and symptoms including anxiety, headache, muscle-twitching, tremor, nausea, and vomiting | Sedation, acute psychosis, psychologic dependence, suicide attempt during intoxication (N=3), violence during intoxication (N=2)  . | Methaqualone, alcohol, cannabis | All subjects were hospitalized: a number of 15 overdose cases required supportive treatment (gastric lavage, IV fluids and respiratory assistance); psychosis treatment consisted antipsychotic agents, such as chlorpromazine, IV fluids, and close observation; withdrawal symptoms required chlorpromazine or diazepam, and in some cases diphenylhydantoin | All patients were U.S. Army Soldiers in West Germany that abused of Mandrax® (DPH + Methaqualone) |
| Promethazine | | | | | | | | | | | |
| Clatts et al., 2010 | Prospective study | N= 136 (on a total of 179) heroin injectors with lifetime use of promethazine | Mean age= 21.3yrs | SUD (opioid) | Bloodborne infections (hepatitis) | IV | NR | Non-medical use of injective promethazine as a substitute for heroin (when heroin is not available or too costly) or to augment the effects of an inadequate heroin dosing (delaying onset of heroin withdraw) | Benzodiazepine, cannabis, amphetamine | NR |  |
| Jensen et al., 2016 | Retrospective study | DPIC dataset N=352, SSI dataset N=204 | Adults (age range 17-60yrs), F | NR | NR | NR | NR | Abuse and suicide were the most recorded intentional causes of promethazine exposures | Antidepressants, antipsychotics, analgesics, recreational drugs/alcohol, others | The outcome varied according to the cause (intentional/accidental) of the exposure, the type of antihistamine drugs (first-/second-generation), and the co-ingestion of other substances and ranged from mild to severe and life-threatening outcomes, requiring hospitalization | The study focused on poisoning patterns from 2007 to 2013 in Denmark based on two independent databases, the Danish Poison and Information Centre (DPIC), and three registers used within the State Serum Institute of Denmark (SSI) |
| Page et al., 2008 | Case series | N=57 promethazine poisonings identified from a prospective database of poisoning admissions (Jan 1987-May 2007) to a regional toxicology service (M: 15, F: 42) | Median age= 22 yrs (17–31) | NR | NR | The median dose  ingested was 625 mg (350–1250 mg) | Tachycardia (HR>100) (56%), hypotension, seizures and CNS depression symptoms | Delirium (42%) | Alcohol, benzodiazepines | Ten of them were admitted to the intensive care unit and four were ventilated; charcoal administration within 2 h appeared to reduce the  risk of delirium occurring both in patients taking promethazine alone and those co-ingesting other drugs; benzodiazepines with or without an antipsychotic were administered in case of drug-induced delirium |  |
| Scott et al., 2007 | Case report | N=1 (F) | Age= 14 yrs | Depression | Migraine | 1,150mg (46 tablets) of promethazine, oral | Low consciousness, tachycardia, generalized increased tone, mydriasis, myoclonic jerks | Delirium with unintelligible speech and distressing visual hallucinations, psychomotor agitation, confusion | Cyproheptadine | Admitted for a drug overdose to the ED, charcoal was withheld and then IV benztropine administered. Psychosis was managed with olanzapine and discharged after nine days | She had been receiving fluvoxamine 150 mg daily |
| Tan et al., 1988 | Case report | N=1 (M) | Age= 25 yrs | NR | Chronic tonsillitis | He developed dependence after two-yrs use, continuing taking the cough mixture due to withdrawal symptoms, increasing dosage, oral | He experienced restlessness, agitation, and abdominal discomfort after abruptly stopping the chronic use | Chronic abuse with addiction and withdrawal symptoms | None | Treated with chlormethiazole to alleviate withdrawal. Low co-operation with the treatment. Referred to a private organization for further treatment | He was referred to a psychiatrist after his arrest for breaking into a hospital dispensary whilst attempting to steal promethazine compound mixture |
| Tsay et al., 2014 | Retrospective review | N= 354 promethazine  abuse and intentional misuse cases reported to the National Poison Data  System (N=95 promethazine alone;  N=259 promethazine in coformulation) | The sample considered 10 yrs and older subjects: exposures were prevalent  among 10 to 19 yrs old and young adults (20s) | NR | NR | Oral | Drowsiness, tachycardia, dizziness, vomiting, hypertension, hypotension, ataxia, and dystonia, respiratory depression and hyperthermia, seizures, and coma | Agitation, confusion, slurred speech, and hallucinations | The most frequent co-formulates were codeine and DXM | Promethazine alone abuse were mostly managed in health care facilities, while promethazine in coformulation had more severe outcomes, requiring ED care management. Outcomes for both cases were up to moderate effects, and there were no reported deaths due to promethazine |  |
| Pseudoephedrine | | | | | | | | | | | |
| Alevizos, 2003 | Case report | N=1 (F) | Age=45 yrs | None | None | Started from 26.25 mg/day and increased to 80mg/day, oral | Decreased appetite, dry mouth, slight tremor, palpitations | She started taking the drug to lose weight and decrease appetite and continued the use for years, needing to increase the daily dosage and developing withdrawal symptoms. Up to 52.50mg/day: euphoria, insomnia, diminished sense of fatigue, and accelerated thinking; at 80mg/day dosage: psychotic symptoms with auditory and visual hallucinations, persecutory delusions, fear, disorganized behaviour; withdrawal symptoms included: dysphoria, restlessness, impaired memory, bulimia, abnormal perceptions | None | She was hospitalized and treated with haloperidol 20 mg/day and thioridazine 150 mg/day. Psychotic symptoms remitted rapidly, but after the discharge from the hospital she experienced depressive symptomatology with suicidal thoughts, which was remitted with clomipramine 100 mg/day. Many psychotic relapses concomitantly to the re-use of the drug. She failed to return to baseline functioning | The patient started taking the drug to lose weight and decrease appetite |
| Diaz et al., 1973 | Case report | N=1 (F) | Age=37 yrs | Depression | None | 3,000-4,500mg of pseudoephedrine/day, oral | Withdrawal symptoms included: slowed speech and psychomotor retardation, tachycardia, tachypnea, insomnia, concrete verbalization | She used the drug for experiencing euphoria and energy. Withdrawal symptoms derived from the abrupt interruption included fatigue, depression, illusions, and hallucinations | None | Admitted to the psychiatric unit and treated for withdrawal symptoms | Initially prescribed for sinus problems, then she increased the dosage and developed chronic use and dependence |
| Koksal et al., 2011 | Case report | N=1 (M) | Age=29 yrs | NR | NR | IV for ten yrs | Speech disturbance, difficulty walking, mask-like face, bradykinesia, symmetric rigidity of legs and marked gait disturbances, twisting feet, postural instability | NR | Acetylsalicylic-acid and potassium permanganate | The subject was treated with fluoxetine 20 mg per/day, amitriptyline 25 mg/day, diazepam 5 mg/day, L-Dopa 225 mg/ day. He was followed up for two yrs without any improvement, and L-Dopa therapy was stopped | “Russian Cocktail”: pseudoephedrine, potassium permanganate, and acetylsalicylic acid diluted in 2 ml of water |
| Leighton, 1982 | Case report | N=1 (M) | Age=27 yrs | Bipolar disorder | None | Abuse of Actifed® for many yrs (100-200ml) at weekends to help relax. Two weeks before increased  intake to 200ml a day, oral | None | Paranoid symptoms, with ideas of influence and auditory hallucinations | None | Presented at the psychiatric outpatient department, lithium treatment was continued as before and trifluoperazine 5 mg nightly added | On lithium treatment |
| Pugh and Howie, 1986 | Case report | N=1 (F) | Age=21 yrs | Depression | None | 50-300ml/day of Actifed®, oral | None | Psychotic symptoms, with auditory and visual hallucinations | None | Treated with oral and depot phenothiazines in addition to supportive psychotherapy | She was convicted of the theft of a medical prescription pad and for making a prescription for herself for Actifed® |
| Sikk et al., 2007 | Case series | N=4 (M) | Mean age= 32,75 yrs (SD=6,38) | SUD (alcohol, cannabis, and heroine) | None | Long-term use of pseudoephedrine and potassium permanganate IV | Disturbance of gait and balance, generalized dystonia, hypokinesia and postural instability, slurred and hypophonic speech | NR | None | NR | This syndrome is similar and probably  identical to manganism (manganese-induced  damage primarily occurs in pathways post- synaptic to the nigrostriatal dopaminergic system) |
| Sullivan, 1996 | Case report | N=1 (M) | Age=18 yrs | Depression | None | 60mg of pseudoephedrine, IV | None | Psychomotor agitation, paranoid delusions, visual and somatic hallucinations, fear | None | Admitted to the psychiatric ward and treated with IM Clopixol® (50mg) and made a complete recovery within 24 hours | Pseudoephedrine was prepared by scraping off the coating, crushing the tablet and dissolving it in water |
| Chlorpheniramine, codeine phosphate, methyl ephedrine and caffeine (BRON®) | | | | | | | | | | | |
| Ishigooka et al., 1991 | Survey | N=44 (M: 32, F: 12) | Mean age: 25.3 yrs | None | NR | Oral | Withdrawal symptoms recorded were perspiration; shivering; headache; diarrhea; vomiting; thirst; heart palpitations; buzzing; convulsion; loss of appetite | Group A: hallucinatory-paranoid state¹ was associated with short usage, relatively small BRON usage term, and little or no physical dependence (few withdrawal symptoms). Group B: affective disorder group² showed larger usage amount, longer usage term, high physical dependence, and autonomic disorders during withdrawal | None | Accessed to mental institutions and rehabilitation centers for substance abuse | ¹Symptoms included: delusional perception, hallucinations, persecutory delusions, and psychomotor excitement; ²symptoms included: depression, irritability, anxiety, and emotional disturbance |
| Murao et al., 2008 | Case report | N=1 (M) | Age= 35 yrs | None | NR | More than 12 tablets daily, oral | Miosis, tachycardia (113 bpm), hypertension (143/ 94mmHg), generalized convulsion, acidosis | Confusion | None | Treated with IV diazepam for the convulsion and IV midazolam continuously for sedation |  |

**TABLE 1. Overview of literature cases of over-the-counter drugs (e.g., antihistamines, cough medicines and decongestants) misuse: summary of the main findings**

ADHD: attention deficit and hyperactivity disease; BP: blood pressure; CNS: central nervous system; CWE: cold water extraction; DH: Dimenhydrinate; DLPFC. L: left dorsal lateral prefrontal cortex; DPH: ddiphenhydramine; DPT: Drug provocation tests; DXM: dextromethorphan; ED: Emergency department; EEG: electroencephalogram; F: female; HC: Healthy Controls; HR: hearth rate; IPL. L: left inferior parietal lobe; ITC.R: right inferior temporal cortex; IV: intravenous; LOC. R: right lateral occipital cortex; lOFC. R: right lateral orbitofrontal cortex; LSD: lysergic acid diethylamide ; M: male; MRI: Magnetic Resonance Imaging; N/A: not applicable; NMDA: N-methyl-D-Aspartate; NR: not reported; OTC: over-the-counter; PreC: bilateral precuneus; PreCG. R: right precentral gyrus; PTSD: post-traumatic stress disorder; ROA: route of administration; RR: respiratory rate; SC: subcutaneous; SD: Standard Deviation; SPECT: Single-photon emission computed tomography; SUD: Substances Use Disorder; TTG: right transverse temporal gyrus.
